# “In Mathematical Language”: On Mathematical Foundations of Quantum Foundations

**DOI:** 10.3390/e26110989

**Published:** 2024-11-18

**Authors:** Arkady Plotnitsky

**Affiliations:** Literature, Theory, and Cultural Studies, and Philosophy and Literature Program, Purdue University, West Lafayette, IN 47907, USA; plotnits@purdue.edu

**Keywords:** continuity, discontinuity, Galois theory, mathematical complexity, reality without realism (RWR) renormalization

## Abstract

The argument of this article is threefold. First, the article argues that from its rise in the sixteenth century to our own time, the advancement of modern physics as mathematical-experimental science has been defined by the invention of *new mathematical structures.* Second, the article argues that quantum theory, especially following quantum mechanics, gives this thesis a radically new meaning by virtue of the following two features: on the one hand, quantum phenomena are defined as essentially different from those found in all previous physics by *purely physical features*; and on the other, quantum mechanics and quantum field theory are defined by *purely mathematical postulates*, which connect them to quantum phenomena strictly in terms of probabilities, without, as in all previous physics, representing or otherwise relating to how these phenomena physically come about. While these two features may appear discordant, if not inconsistent, I argue that they are in accord with each other, at least in certain interpretations (including the one adopted here), designated as “reality without realism”, RWR, interpretations. This argument also allows this article to offer a new perspective on a thorny problem of the relationships between continuity and discontinuity in quantum physics. In particular, rather than being concerned only with the discreteness and continuity of quantum objects or phenomena, quantum mechanics and quantum field theory relate their continuous mathematics to the irreducibly discrete quantum phenomena in terms of probabilistic predictions while, at least in RWR interpretations, precluding a representation or even conception of how these phenomena come about. This subject is rarely, if ever, discussed apart from previous work by the present author. It is, however, given a new dimension in this article which introduces, as one of its main contributions, a new principle: the mathematical complexity principle.

## 1. Introduction

A brief explanation of my terminology and abbreviations is in order before I proceed. Physics as a science involves both establishing physical phenomena themselves, in which case one commonly refers to experimental physics, and investigating these phenomena by theoretical means, in which case one commonly refers to theoretical physics. Both relativity theory, special and general (hereafter GR), and quantum mechanics (QM) and quantum field theory (QFT) are parts of theoretical physics, within, respectively, relativistic and quantum physics. GR, QM, and QFT are standard abbreviations for these theories and they are adopted here. The case is a bit trickier with “classical physics”, the term introduced in the twentieth century to distinguish it from “new physics”, relativistic or quantum. The term “classical physics”, however, also commonly refers to classical physical theories, such as classical mechanics, classical statistical physics, or classical electromagnetic theory. I retain this use, qualifying it when necessary to avoid ambiguity. 

The argument of this article is threefold:

(1) The article argues that from its rise in the sixteenth century in the work of such figures as Galilei, René Descartes, and Johannes Kepler to our own time, the advancement of modern physics as a mathematical-experimental science has been defined by the invention of *new mathematical structures,* possibly borrowing them from mathematics itself. Among the greatest such inventions (all using differential equations) are:

* Classical mechanics based on analytic geometry and calculus by Sir Isaac Newton;

* Maxwell’s electromagnetic theory based on the concept of classical field and its mathematization as represented by Maxwell’s equations; 

* Relativity theory, special and general theory (GR) based on Riemannian geometry;

* Quantum theory, ultimately as QM and QFT, based on the mathematics of Hilbert spaces over C and the operator algebras. I refer to the standard version of quantum formalism introduced by John von Neumann and still most commonly used, rather than the original ones of Werner Heisenberg’s matrix mechanics, developed by Max Born and Pasqual Jordan, Erwin Schrödinger’s wave mechanics, and Paul Dirac’s q-number QM. There are still other versions of the formalism, such as C*-algebra and category–theory ones. All these versions are essentially equivalent mathematically.

(2) This article argues that QM and QFT (to either of which the term “quantum theory”, unless qualified, refers hereafter) gave this thesis a radically new meaning by virtue of the following two features:
  (a) On the one hand, quantum phenomena themselves are defined by *purely physical features* as essentially different from all previous physics, beginning with the role of Planck’s constant *h* in them, features manifested in such paradigmatic quantum experiments as the double-slit experiment or those dealing with quantum correlations.
  (b) On the other hand, quantum theory qua theory, at least QM or QFT, is defined, by *purely mathematical postulates*, which connect it to quantum phenomena strictly in terms of probabilities of predicting the data observed in these phenomena without requiring that quantum theory represent how these phenomena physically come about.

These two features may appear discordant, if not inconsistent, especially given that previously, in both classical physics and relativity theory, the physical features of the corresponding phenomena and mathematical postulates defining these theories were connected, *representationally*, by mathematically representing how the phenomena considered come about by the corresponding theory. I argue, however, that (a) and (b) are in accord with each other, at least in certain interpretations (including the one adopted here), designated as “reality without realism”, RWR, interpretations, introduced by this author previously (e.g., [1,2,3]). In Heisenberg’s invention of QM, moreover, and then in Dirac’s invention of his version of QM and quantum electrodynamics (QED), physics emerges from mathematics rather than making mathematics of a theory follow physics, as was the case in theoretical physics previously.

(3) The argument outlined in (2) above allows this article to offer a new perspective on a thorny problem of the relationships between continuity and discontinuity in quantum theory. Quantum theory, along with quantum physics, was introduced with this problem at the core with Max Planck’s discovery of his black-body radiation law in 1900 as a physics of discontinuity, specifically as discreteness, to which the term “quantum” originally referred. In particular, rather than being concerned only with the discreteness and continuity of quantum objects or phenomena (which are essentially different from quantum objects), QM and QFT relate their continuous mathematics to the irreducibly discrete quantum phenomena in terms of probabilistic predictions. At the same time, QM and QFT in RWR interpretations preclude a representation or even conception of how these phenomena come about, in accord with (2) above. As a complex combination of geometry and algebra, this mathematics contains discrete structures as well, but so does the continuous mathematics of classical physics and relativity theory. The point here is the fundamental, irreducible role of continuous mathematics in QM and QFT. This subject is rarely, if ever, discussed apart from previous work by the present author [1,2,3,4]. It is, however, given new dimensions in this article in connection with QFT and renormalization. The fundamentally probabilistic nature of quantum theory is fully in accord with the experimental evidence, available thus far, because no other predictions than probabilistic are in general possible in quantum experiments.

These three lines of this article’s argumentation are interconnected, supporting and even defining each other, especially (2) and (3). 

Modern physics emerged in the work of Descartes, Kepler, Galileo, and others, along with modernity itself. Physics became a mathematical–experimental science that deals with the material constitution of matter in fundamental physics, such as classical mechanics or classical electromagnetic theory, relativity theory, and quantum theory, with the ultimate constitution of matter. Although this is not always sufficiently recognized, mathematics governed this conjunction beginning with the rise of modern physics, initially the mathematics of geometry. Thus, Newton was still compelled to use geometry in presenting his new mechanics, discovered by him by means of calculus (not considered legitimate mathematically at the time), in *Philosophiae Naturalis Principia Mathematica* [5]. As stated above, this primacy of mathematics, reflected in the full title of Newton’s *Principia*, was, however, recognized by Martin Heidegger. In reflecting on the rise of modern physics in these figures, Heidegger observed: “Modern science is experimental because of its mathematical project” [6] (p. 93). While the case requires qualifications as concerns other natural sciences, such as biology, it is straightforward as concerns modern physics. That does not mean that modern physics does not contain aspects that are not mathematical. It is mathematics, however, that *defines* modern physics as a science, even experimental physics. 

This is the case not only or even primarily because of the role of measurement in it vs. Aristotle’s physics as a physics of qualitative observations, although the role of (quantitative) measurement in modern physics is essential, including vis-à-vis Aristotle’s physics. More significant is that modern physics uses mathematics to relate to and especially to predict, quantitatively, the data found in experimentally observed phenomena. This use of mathematics, initially, again, especially geometry, has ancient Greek science as part of its history. Ancient Greek geometry, *geo*-*metry*, was also a science [*episteme*] of measurement of distances of physical surfaces and solid bodies, which made it both physical and mathematical. Modern (and even some ancient Greek) scientists extended geometry to spaces beyond earth, ultimately to the cosmological scale, still defined now as in the post-Big-Bang extending universe geometrically. Ancient *physics*, on the other hand, specifically that of Aristotle, was primarily a qualitative theory of motions of entities, material or mental, in correspondingly physical or mental domains. Modern physics restricted itself to dealing, mathematically, with natural phenomena alone (which is sometimes referred to as “the Galilean reduction”) while also extending its reach to the cosmological scale, thus making the Universe mathematical, specifically geometrical. Galileo famously saw geometry as the language in which the book of nature is written. 

The main twentieth-century incarnation of a fundamentally *geometrical* view of cosmology came with Albert Einstein’s GR (introduced in 1915). GR made the physics of gravity a geometry by using modern mathematics, Riemannian geometry. The theory was quickly (around 1917) applied by Einstein to cosmology and has shaped the cosmological thinking in theoretical physics since. By 1915, however, *modern* geometry was largely separated, abstracted from physics, a defining aspect of modern mathematics. Einstein returned this mathematics to physics, a move repeated epistemologically more radically (on RWR lines) by Heisenberg in his invention of QM. In the case of the mathematics of spatiality, this separation of modern mathematics from physics was amplified by the role of topology, a modern discipline, although it has an earlier genealogy, in particular in the work of Leonhard Euler. Euler was one of the key figures, along with Jean-Baptiste le Rond d’Alembert and Pierre Simon de Laplace, in the history of the relationships between mathematics and physics in the eighteenth century, before the emergence of modern mathematics defined by its separation from physics. At the same time, however, Euler’s work on topology indicates that the situation is more complex as concerns both separating mathematics from physics and connecting, or reconnecting it, to physics. This complexity is also found in the work of such major representatives of modern mathematics as Karl Friedrich Gauss, Riemann, and Henri Poincaré in the nineteenth century, and David Hilbert, Hermann Weyl, and John von Neumann in the twentieth century, with Poincaré crossing into the twentieth century. Nevertheless, the move of returning modern mathematics, previously abstracted from physics, was pronounced in both relativity theory and, epistemologically more radically, QM and QFT.

The most fundamental reason for Heidegger’s claim that modern physics is experimental because of its mathematical project was, then, that from its emergence on, modern physics uses mathematics to relate to and especially to predict the quantitative data found in experimentally observed phenomena. This is manifestly the case in classical physics, relativity theory, and quantum theory, the main types of fundamental theories (theories dealing with the ultimate constitution of matter) now, with several theories comprising each. Classical physical theories, especially classical mechanics, are not always seen as fundamental theories, but for the reasons explained below and in detail in [7], they may be and are in the present article. René Thom even argued that mathematics already played an analogous role in Aristotle’s physics, which Thom saw as *in effect* containing (qualitative) topology [8,9,10]. The main difference would then be that, unlike Aristotle’s qualitative topology of physics, the mathematics of modern physics is also quantitative, as is required in physics if it needs to relate to measurements of the quantitative data observed in phenomena. That, however, does not invalidate but only qualifies the claim that from Aristotle on, physics is defined by mathematics, initially geometry or even proto-topology, although the Pythagoreans defined their physics or at least their cosmology by both geometry and arithmetic. The Pythagorean harmony of the cosmic spheres was represented by proportions or, in our terms, rational numbers, the cosmology, and this mathematical thinking itself, shattered by the Pythagoreans’ discovery of the incommensurable magnitudes, such as those of the diagonal and the side of a square. Geometry became the dominant form of mathematical thinking and the primary mathematical model for philosophy.

Beginning with the mathematics emerging at the rise of modernity, roughly around 1600, mathematics restored the role of both geometry and (by then) algebra to physics and made physics modern. This is how we still see the universe now, by extending the representation of its geometry and topology from the Riemannian spaces, accompanied by the tensor calculus on them, of GR to such stratospheric objects as Calabi–Yau manifolds or Alexandre Grothendieck’s motive theory. While the expanding macroscopic space of the universe appears to be on average flat, its origin in the Big Bang or what happened before it is a separate matter, especially given that this early history may be quantum in nature. If so, it may not be possible to speak or even conceive of its ultimate constitution, including as either continuous or discrete.

In dealing with the interpretation of quantum theory, one cannot avoid the question of the nature of reality, both material and, because of the mathematics of quantum theory, mental, and of our capacity to deal with either reality by representing, knowing, or conceiving of it, or the impossibility thereof. “Reality” is assumed here to be a primitive concept and is not given an analytical definition. By “reality” I refer to that which is assumed to exist, without making any claims concerning the character of this existence, claims that, as explained below, define realism, or in the case of mathematics, Platonism (although “mathematical realism” is used as well). On the other hand, the absence of such claims allows one to place this character beyond representation or, which is the view assumed in this article, even beyond conception. This placement is considered here under the heading of reality without realism (RWR) following [1,2]. I understand existence as a capacity to have effects on the world. The assumption that something is real, including of the RWR-type, is made, by inference, on the basis of such effects as experiential phenomenal effects, rather than something as merely imagined. By the same token, RWR interpretations allow for and even require a representation of these effects but not a representation or even a conception of how they come about. As RWR, the ultimate reality that makes them possible cannot be experienced as such, but only makes possible the assumption of its existence through these effects. 

It is more rigorous to see a different interpretation of a given theory as forming a different theory, because an interpretation may involve concepts not shared by other interpretations. For simplicity, however, I will continue to speak of different interpretations of QM or QFT. By contrast, in the case of classical physics and relativity theory, as is common, I refer to theories themselves. This is because most interpretations of these theories, including the ones assumed here, are realist or (which is another common term for such theories) ontological. It is worth keeping in mind that, just as classical and quantum physics, relativity is not restricted to theories, and I qualify when I refer to relativistic phenomena rather than special or general relativity theory. I also use the term “view” to indicate a broader perspective grounded in a given interpretation, such as the realist view vs. the RWR view.

The concept of RWR arguably originated in the foundations of mathematics. It was suggested by Henri Lebesgue, one of the founders of modern integration and measure theory, in the wake of the paradoxes of Georg Cantor’s set theory [11]. The idea has remained marginal and rarely, if ever, adopted in mathematics [2]. The idea has been better known, if debated, in fact always remaining a minority view, in physics following QM, especially in Niels Bohr’s RWR interpretation of it, the type of interpretation adopted here along with the designation itself following [1,2]. (Bohr did not use the designation “reality without realism”.) RWR interpretations place the *ultimate reality* responsible for quantum phenomena beyond conception. At the same time (hence, my emphasis), these interpretations assume that such a conception or even representation is possible in considering quantum phenomena as observed phenomena, which are effects of this ultimate reality. The existence of this reality is inferred from these effects, predicted by the mathematics of QM. In the case of classical physics and relativity theory, a mathematical, in fact (while involving algebra and analysis, in this case commutative) geometrical, representation is possible at all levels of reality considered. While it was fundamentally defined by new (noncommutative) algebra of Hilbert-space operators, QM also introduced into physics a new form of geometry, that of Hilbert spaces over C, in which spaces these operators functioned. The mathematics already existed in mathematics itself. The geometry of Hilbert spaces was one of Hilbert’s many contributions to geometry, although usually seen as that to functional analysis, which it was as well. Initially, QM did not use this formalism, and it was translated into, in fact axiomatized in, these terms by von Neumann [12], who also introduced the term Hilbert space in mathematics. The concept of a Hilbert space was invented by Hilbert, who did not define it in purely abstract terms as von Neumann did. 

The assumption of the independent existence of nature or matter essentially amounts to the assumption that it existed before we existed and will continue to exist when we no longer exist. This assumption has been challenged, even to the point of denying that there is any material reality vs. mental reality, with Plato as the most famous ancient case and Bishop Berkeley as the most famous modern case. Such views are useful in suggesting that any conception of how anything exists, or even that it exists, including as independent of human thought, belongs to thought. It need not follow, however, that something which such concepts represent or to which they relate otherwise than by representing it, possibly placing it beyond representation or even conception, does not exist, including as a form of material reality. In any event, that any conception of how anything exists or even that it exists (including as beyond thought) still belongs to thought need not imply that something beyond the reach of thought does not exist. 

This was indeed Lebesgue’s point made in 1905. This was two decades before QM, which, introduced in 1925–1926 by Heisenberg and Schrödinger, brought the RWR view into the debate concerning fundamental physics. Quantum theory, discovered by Planck in 1900, was barely introduced by the time of Lebesgue’s comment. Whether Lebesgue knew about it or not, it could not have been his source, because it had not posed this type of possibility then. Bohr’s 1913 atomic theory used the RWR view in dealing with the “quantum jumps” (transitions between stationary, constant-energy states of electrons in atoms) still a decade after Lebesgue’s comment. In commenting on the paradoxes of set theory, shaking the foundations of mathematics then, Lebesgue noted that the fact that we cannot imagine or mathematically define objects such as “sets” that are neither finite nor infinite does not mean that such objects do not exist [11] (pp. 261–273) [13] (p. 258). Lebesgue did not specify in what type of domain, material or mental, such entities might exist. His observation is, however, a profound reflection of the possible limit of our thought concerning the nature of reality, material or mental. Similarly, the fact that we cannot conceive of entities that are neither continuous nor discontinuous does not mean that such entities do not exist, including in nature, a possibility brought about by quantum phenomena. Indeed, Lebesgue’s insight was also a response to the problem of the continuum and the debates concerning it shaped by Cantor’s work and especially his continuum hypothesis. The latter was given a deeper and more radical understanding by Gödel’s incompleteness theorems of 1931 and then Paul Cohen’s proof of the undecidability of the continuum hypothesis in the 1960s. The undecidability of Cantor’s continuum hypothesis suggests that the concept of continuum may not be realizable mathematically. The possibility that the continuum does not exist in nature, for example, as space, time, or motion, has been considered long before then, even by the pre-Socratics.

It need not follow, however, that the ultimate constitution of physical reality, spatial or temporal, is discrete, although this view is possible and has been proposed. Instead, it may be beyond anything that we can conceive, and hence no more discrete than continuous, in short, RWR, which it is assumed to be in RWR interpretations. On the other hand, in RWR interpretations, reality is assumed to exist in nature, in accord with Lebesgue’s observation that something that cannot be thought by us can, nevertheless, exist as real. As Lebesgue appears to have understood as well, it is equally impossible to be certain that any such reality does exist. The possibility of their existence, however, reflects both possible limits of our thought and our thought’s capacity to conceive of this possibility. While not originating in the RWR view, Gödel’s incompleteness theorems and the undecidability of the continuum hypothesis are open to and may even entail this view of mathematical reality as a mental reality [2].

Assuming this type of reality, even if only with a practical justification (no other justification is claimed in this article or is possible in the RWR view), is a philosophical wager. It was, however, this wager that led Heisenberg to QM, defined by a new mathematics combining both continuity and discreteness, and correlatively geometry and algebra, the mathematics enabling QM to predict quantum phenomena, irreducibly discrete relative to each other. Given the nature of the ultimate reality responsible for quantum phenomena as beyond representation or even conception, it is not surprising that, in accord with the second line of my argument, QM or QFT is defined, including as different from especially classical physics, by *purely mathematical postulates*, which connect either theory to quantum phenomena strictly in terms of probabilities. As explained below, it follows that under these conditions, these predictions could only be probabilistic, which is, as noted, strictly in accord with quantum experiments. By the same token, the nature of quantum probability is different from that of classical physics like classical statistical physics or chaos theory. There, the recourse to probability is a practical, epistemological matter. It is due to our lack of knowledge concerning the underlying behavior of the complex systems considered while their elementary constituents could in principle be predicted exactly deterministically. In the case of quantum systems, in RWR interpretations, there is no possible knowledge or even conception concerning the behavior of a quantum system, no matter how simple. 

The circumstances just outlined change the nature of causality in quantum theory, compelling me to distinguish between “classical causality” and “quantum causality”. By “classical causality”, commonly called just “causality”, I refer to the claim that the state *X* of a physical system is determined, in accordance with a law, at all future moments of time once its state *A* is determined at a given moment of time, and state *A* is determined by the same law by any of the system’s previous states. This assumption implies a concept of reality which defines this law, thus making this concept of causality realist or ontological. Some, beginning with P. S. Laplace, have used “determinism” to designate classical causality. I prefer to use “determinism” as an epistemological category referring to the possibility of predicting the outcomes of classically causal processes ideally exactly. In classical mechanics, when dealing with individual or small systems, both concepts are co-extensive. On the other hand, classical statistical mechanics or chaos theory are classically causal but not deterministic in view of the complexity of the systems considered, which limit us to probabilistic or statistical predictions concerning their behavior. The main reason for my choice of “classical causality”, rather than just causality is that, in view of the nature of quantum probability in RWR interpretations, it is possible to introduce alternative, probabilistic concepts of causality applicable in QM, at least in RWR interpretations, where classical causality does not apply. This concept is considered, under the heading of “quantum causality”, in [1] (pp. 207–218).For the present purposes, it suffices to state its basic definition: An actual quantum event A allows one to determine and predict which events may happen with one probability or another but, in contrast to classical causality, without assuming that any of these events will necessarily happen, regardless of outside interference (an outside interference can change what can happen when classical causality applies, while, however, restoring classical causality after this interference). As Bohr stressed:

[I]t is most important to realize that the recourse to probability laws under such circumstances is essentially different in aim from the familiar application of statistical considerations as practical means of accounting for the properties of mechanical systems of great structural complexity. In fact, in quantum physics we are presented not with intricacies of this kind, but with the inability of the classical frame of concepts to comprise the peculiar feature of indivisibility, or “individuality”, characterizing the elementary processes [14] (v. 2, p. 34).

The “indivisibility” refers to the indivisibility of phenomena in Bohr’s sense, defined by the impossibility of considering quantum objects independently of their interactions with these instruments, as explained in detail below. “Individuality” refers to the assumption that each phenomenon is individual and unrepeatable, is the outcome of a unique act of creation, discrete relative to any other phenomenon while also embodying the essential randomness of quantum physics. This kind of randomness, quantum randomness, is not found in classical physics. This is because even when one must use probability in classical physics, at the bottom, one deals with individual processes that are classically causal and in fact are at the bottom deterministic and in the first place allow for a (realist) representation by means of classical mechanics. There are situations such as those of the Einstein–Podolsky–Rosen (EPR) type of experiments where predictions concerning certain variables are ideally possible with probability equal to one. It can, however, be shown that, in view of the conditional nature of these predictions, they still do not entail classical causality [1] (pp. 207–218).

It follows, in accord with the third line of my argument, that if the ultimate nature of the reality responsible for quantum phenomena cannot be assumed to be either continuous or discrete, there is no special reason to assume that the mathematics required to predict the data observed in quantum phenomena should be discrete or even finite, as some argue to be necessary, rather than continuous. The reasons for this argument are most commonly the residual problems of QFT, dealing with the appearance of mathematical infinities and divergent objects, such as certain integrals, related to renormalization. These infinities disappear if the mathematics used is finite. In the present RWR view, this mathematics could be either, insofar as it makes correct predictions concerning the phenomena considered which are always discrete relative to each other and are represented by classical physics. I argue, however, that the nature of quantum phenomena imposes requirements on this mathematics if it is discrete (including finite). Specifically, this mathematics must have a structural complexity analogous and related to that of the continuous mathematics that has been used in quantum theory thus far. This view is one of the main contributions of this article, under the heading of *the mathematical complexity principle*. 

The article proceeds as follows. The next section deals with classical physics and relativity theory defined as continuous physics by their mathematical projects. It also revisits Aristotle’s physics with which modern physics, in particular in the case of classical physics and relativity theory, are still linked by virtue of realism, continuity, and causality. Section 3 and Section 4 consider the mathematical–experimental architecture of quantum theory, specifically QM and QFT, focusing on its experimental side in Section 3 and mathematical side in Section 4. Section 5 deals with the relationships between continuity and discontinuity in quantum theory and fundamental physics in general. My main argument is that, while experimentally defined by discontinuity, QM or QFT are mathematically defined by continuous mathematics, although they do contain discrete mathematical structures. (That, however, is also true about classical physics and relativity theory.) I then consider the question of discrete quantum theories in high-energy regimes governed by QFT in relation to renormalization. 

## 2. The Physics of Continuum from Aristotle to Einstein and Beyond 

I begin with the definition of observation and measurement in physical theory via Bohr’s remark. Bohr was commenting on quantum experiments. In effect, however, he was also establishing, along with distinguishing aspects of quantum observations and measurement, a continuity between the concepts of observation and measurement in all modern physics (classical, relativistic, and quantum) and even, as concerns *observation*, Aristotelian physics. In Aristotle’s physics, there was no measurement, at least not as the main ingredient of physics, because some elements of measurement could be found there. Modifying Bohr’s formulation to bring out this continuity (with the new features of quantum physics considered below), the following formulation is obtained:

Any measurement in a physical theory refers either to a fixation of the initial state, or an initial preparation, at t_0_ or to the test of such predictions, predicted observations, at t_1_, and it is the combination of measurements of both kinds which constitutes a well-defined physical experiment [15] (p. 101; paraphrased).

One must refer to a physical *theory*, because, as Bohr noted elsewhere, while “ultimately, every observation can, of course, be reduced to our sense perceptions, … in interpreting observations use has always to be made of theoretical notions” [14] (v. 1, p. 54). In QM, these notions include those defining quantum phenomena, specifically the role of *h* in them, which leads to other new features of these phenomena vis-à-vis classical physics and relativity. (On that earlier occasion, in 1927, Bohr expressly refers, under the heading of quantum postulate, to the role of *h* as “symbolizing” the nature of quantum phenomena, the subject considered in [1] (pp. 171–174) [16].) In fact, the above definition applies even in Aristotelian physics with which classical physics retains more proximities than it is often thought, although the fact that in Aristotle’s physics one deals only with observation rather than measurement remains crucial. It may indeed be instructive to comment on Aristotle’s physics first. I use the following diagram, hereafter Diagram A:

E_0_ (q_0_)-------------------------------------------→ E_1_ (q_1_) t_0_                   t_1_

This diagram as such applies in all physics considered here. What changes are the concepts involved and connections between them, which also indicates that diagrams are rarely sufficient and may, if their concepts are not properly considered, be misleading. In Aristotle’s physics, events E_0_ and E_1_ of observing positions q_0_ and q_1_ are assumed to be connected by a continuous, classically causal process represented qualitatively to our phenomenal intuition. This representation defines observations, in accordance with (in our language) the topology of continuity and, in the case of some motions, such as those of projectiles, geometry of straight lines. Thus, as noted, there is still some mathematics defining physics. 

Classical physics changed, and in several key respects corrected, Aristotle’s physics. The nature of these corrections is well known and need not be rehearsed here. My concern is the conceptual (mathematical–experimental) transformation enacted by modern physics, which, however, retains connections to Aristotle’s physics on the account of continuity and causality. This transformation changes the meaning of Diagram A, which still applies as a diagram.

E_0_ (q_0_)-----------------------------------------------→ E_1_ (q_1_) t_0_                   t_1_

Events E_0_ and E_1_ of observing and quantitatively measuring coordinates q_0_ and q_1_, respectively, are still assumed to be connected by a continuous causal process, but now represented mathematically. This representation may be seen as a mathematical refinement of the general phenomenal representation of bodies and motions in space and time, the representation on which Aristotle’s physics is based, refining this representation only philosophically and essentially qualitatively.

Galileo was arguably the most important figure in establishing modern physics as a mathematical–experimental science while also granting geometry the main, including cosmological, significance in physics. This significance has not been diminished by calculus, which introduced new algebraic dimensions into physics. Algebra was far from absent in Galileo or Descartes. Geometry, however, a heritage of ancient Greek mathematics, remained dominant, not the least as the way of *thinking* about physics, including in Newton, his use and the very invention of calculus notwithstanding. That was only to change with Heisenberg and QM, compelling Einstein to speak, rather disparagingly and not entirely accurately, of Heisenberg’s method as “purely algebraic”. In fact, as explained below, Heisenberg’s “method” also brought in new geometry to physics along with new algebra [1] (pp. 111–126).

Galileo, as Edmund Husserl observed, inherited geometry itself as an already established field, preceding his physics and thus modern physics: “The relatively advanced geometry known to Galileo, already broadly applied not only to the earth but also in astronomy, was for him, accordingly, already pregiven by tradition as a guide to his thinking, which [then] related empirical matters to the mathematical ideas of limit” [17] (p. 25). Galileo’s thinking, however, moved beyond this tradition, including as manifested in thinking of his predecessors such as Copernicus, Descartes, and Kepler (of whose work Galileo was not aware). Galileo’s thinking was bolder in assigning geometry as the science of physical space a cosmological meaning, and reciprocally the cosmos a mathematical, geometrical meaning. This thinking made the cosmos mathematical, or at least mathematically representable. The reason for making this strong claim is that this was not only or primarily a matter of considering the solar system geometrically, which was already the case in his predecessors. Indeed, even apart from the fact that a version of the Copernican system was known to the ancient Greeks, the Ptolemean system was cosmological, too. Galileo’s thinking was much more radical. Galileo replaced philosophy with mathematics, especially geometry (although he also uses functions, and thus algebra), in physics, thus bracketing philosophy, or at least the phenomenological part of it, from physics. In Galileo and in all modern physics after him, the primordial grounding of physics was in mathematics, which was primordially grounded only in itself rather than in philosophy. It was this break with philosophy (in both physics and mathematics) that worried Husserl and was even seen by him as a crisis, extending to our own time [17].

Galileo’s cosmos was a mathematical cosmos, written as “the book of nature” in the language of geometry. On the other hand, the idea of the book of nature, known to his precursors in physics, was borrowed by Galileo from theology. Thus, this new scientific cosmology emerged from two different trajectories or technologies of thought, theological and mathematical, akin to the way present-day smartphones have emerged from and merged two technologies, the telephone and the computer. Galileo’s thinking was more multiply shaped, given the philosophical trajectories of his thinking, extending from ancient Greek philosophy to his own time, and literary and artistic influences on his ideas, such as those of Dante’s poetry or Titian’s paintings. In 1558, on the invitation of the Florentine Academy, Galileo also read two lectures “On the Shape, Location, and Size of Dante’s Inferno”, which secured him a position as a lecturer in mathematics at the University of Pisa (there are also more trajectories to the genealogy of the smartphone). In Galileo’s famous words,

Philosophy is written in that great book which ever is before our eyes—I mean the universe—but we cannot understand it if we do not first learn the language and grasp the symbols in which it is written. The book is written in mathematical language, and the symbols are triangles, circles and other geometrical figures, without whose help it is impossible to comprehend a single word of it; without which one wanders in vain through a dark labyrinth [18] (pp. 183–184).

Galileo thus departs from Aristotle’s physics both in making his physics that of nature alone (“the Galilean reduction”) and in assuming a fundamentally mathematical character of the philosophy written in the book of nature which could not be read without mathematics. In sum, more than anyone else before him, Galileo granted mathematics its defining role in modern physics, a role eventually codified by Newton in *Principia* [5], and made modern physics experimental by making it mathematical as Husserl and, following him, Heidegger argued [6] (p. 93). As noted, a predominantly geometrical view was restored to cosmology by Einstein in GR, which made the physics of gravity a geometry by using modern mathematics (by then developed separately from physics), Riemannian geometry. Maxwell’s electromagnetism, Einstein’s main model, was a key precursor physically, but it was not as expressly geometrical as was Einstein’s GR, which Einstein tried to bring together with electromagnetism for the rest on his life without ever succeeding. While, however, it is fundamentally different, both physically and mathematically, from classical physics, relativity theory, special or general, remains a theory of a continuum, inheriting this aspect of it from classical physics. In the case of special relativity, Diagram A acquires the following meaning.

E_0_ (q_0_) -----------------------------------------------→ E_1_ (q_1_) t_0_                   t_1_

Events E_0_ and E_1_ of observing positions q_0_ and q_1_, respectively, are still assumed to be connected by causal process, but are now represented by the equations of special relativity, including Lorentz’s equation for the addition of velocities. As such, special relativity represents a radical ontological and epistemological transformation of the concept of motion, reflected by the role of *c*, which is a measurable numerical constant. Special relativity is grounded in two main postulates. Postulate 1 is that of Galilean relativity. Postulate 2 is a new postulate, remarkable physically but easily represented mathematically.

1. The laws of physics are invariant in all inertial frames of reference.
2. The speed of light *c* in vacuum is the same for all observers, regardless of the motion of light source or observer.

The difference from classical physics is the second postulate. It becomes part of the first postulate if the laws of physics include Maxwell’s equations. On the other hand, the laws of Newton’s mechanics would, then, no longer apply without modifications, such as Lorentz’s equation for addition of velocities, which mathematically represent the second postulate. This equation was part of the new mathematics of relativity theory. Relativity theory thus posed insurmountable difficulties for our general phenomenal intuition, because the relativistic law of addition of velocities (defined by the Lorentz transformation)

$$s=\frac{v+u}{1+(vu/{c)}^{2}}$$

for collinear motion runs contrary to any possible intuitive conception of motion. We cannot conceive of this kind of motion by our general phenomenal intuition. This makes this concept of motion no longer a mathematical refinement of a daily concept of motion as the concept of motion is in classical physics. It is an independent physical concept that is, however, still represented mathematically, in contrast to QM or QFT in RWR interpretations. There, the ultimate reality considered (responsible for quantum phenomena) is beyond any conception we can form, including a mathematical one. Special relativity is, nevertheless, the first physical theory that defeated our ability to form a visualization of an individual physical process, although the concept of (classical) field in electromagnetism already posed certain complexities in this regard.

GR, in accord with my argument, is a remarkable example, arguably the greatest since Newton’s mechanics, of advancing physics by means of *new mathematical structures* in this case of expressly borrowing them from mathematics itself, Riemannian geometry. It also retained and enhanced epistemological aspects of special relativity as concerns the difficulty and ultimately impossibility of grasping its physics by means of our general phenomenal intuitions, thus only allowing for a mathematical realism.

Bohr did not miss this point. He said: “I am glad to have the opportunity of emphasizing the great significance of Einstein’s theory of relativity in the recent development of physics with respect to our emancipation from the demands of [intuitive] visualization” [14] (v. 1, pp. 115–116). “Emancipation” is not a casual word choice, rarely found in Bohr’s writings. Nevertheless, relativity still offers a mathematically idealized representation of the reality considered, thus making this reality available to thought, even if not our general phenomenal intuition. This is no longer possible in quantum theory, in RWR interpretations, such as that of Bohr (in its ultimate version) or the one assumed here. 

Bohr acutely realized the existence, or at least a possible existence, of a reality beyond the reach of representation or even conception that was, nevertheless, ultimately responsible for what is available to thought and even to our immediate phenomenal perception as quantum phenomena. Bohr saw this new situation in fundamental physics as “an epistemological lesson of quantum mechanics” [14] (v. 3, p. 12). At least, this was an epistemological lesson of his *interpretation*. Perhaps, however, physics cannot teach us its epistemological lessons other than by an interpretation. There appears, however, to be more consensus as concerns the interpretation of classical physics and relativity theory as realist theories, although this is not an entirely unanimous one either. It has been questioned, for example, whether the mathematical architecture of relativity theory corresponds to the architecture of nature, as opposed to merely serving as a mathematical model for correct predictions concerning relativistic phenomena (e.g., [19]). In this case, these predictions are deterministic as opposed to the irreducibly probabilistic predictions of quantum theory, even in dealing with most elementary individual quantum phenomena. This is a fundamental difference due to the impossibility, in principle, of controlling the interference of observational instruments with the quantum object under investigation, regardless of interpretation. When it comes to QM or QFT, the proliferation of diverse (and sometimes incompatible) interpretations and the debates concerning them has been uncontainable and continues with an undiminished intensity and no end in sight. 

## 3. Quantum Discontinuum: “Interactions Between Atoms and the External World” 

Quantum physics brought the “emancipation” from the demands of visualization invoked by Bohr to the level of making the ultimate reality responsible for quantum phenomena “invisible to thought”. The meaning of this expression, introduced in [7], is essentially the same as that of reality without realism. At least in RWR interpretations, quantum theory, specifically QM and QFT, placed this reality beyond the reach of thought, any thought, even mathematical one, rather than only our general phenomenal perception or intuition. Similarly to the role of *c* in special relativity, this situation is reflected in the role of Planck’s constant *h* which is, just as *c*, numerical and experimentally observed. 

Speaking of the ultimate reality responsible for quantum phenomena as “invisible to thought” follows Bohr’s appeal to the impossibility of visualization of this reality. Bohr’s use of the term “visualization” in part owed to the German term for intuition, *Anschaulichkeit*, which etymologically relates to what is visualizable. Bohr, however, assigned it a broader meaning of being available to our general phenomenal intuition. This availability is, as explained, no longer possible in relativity theory, which, however, still allows for a mathematical representation of all levels of reality considered. In Bohr or the present view, no representation or even conception of any kind, including a mathematical one, is any longer possible in the case of the ultimate reality responsible for quantum phenomena. Such a representation is, however, possible in considering quantum phenomena, assumed to be represented by classical physics. We can “see” in our conscious experience what is observed in quantum experiments. We can also consciously experience the mathematics used to predict what is observed. As a result, both can be communicated unambiguously, and in this sense, but in the present view *only in this sense*, can be assumed to be objective. 

At least in RWR interpretations, however, we cannot experience or represent, including mathematically, or even conceive of, *think* how quantum events that we see come about. In other words, we cannot perceive or even conceive of the reality ultimately responsible for what we can phenomenally experience but we see the effects of this reality in our phenomenal experience. Nobody has ever *seen* (no matter how good one’s instruments) a moving electron or photon. One can only see a trace, say, a spot on a silver bromide plate, left as an effect of the interaction between an electron or a photon and the instrument used, capable of this interaction and creating this trace. Quantum physics is a physics of traces, the origins of which can never be reached experimentally. This is the case regardless of interpretation. RWR interpretations, however, in principle preclude any representation or even conception, physical or mathematical, of how quantum phenomena come about. As stated, we also cannot form a general phenomenal conception of the reality considered in relativity theory or the reality of electromagnetic field but only of effects of either reality. Either reality can, however, be mathematically represented and thus made “visible to thought” vs. the ultimate reality responsible for quantum phenomena in RWR interpretations. This reality is not only invisible to our phenomenal perception but is also invisible to thought, is beyond the reach of thought.

In order, however, to avoid the complexities involved in using the term visualization (or the impossibility thereof) which can be associated with different concepts, henceforth, in considering RWR interpretations, I mostly speak of the ultimate reality responsible for quantum phenomena as “beyond conception” or “beyond thought”. Bohr never expressly used these terms as such, but his ultimate interpretation, introduced around 1937, was an RWR interpretation based on this view of the ultimate reality responsible for quantum phenomena. The present interpretation, but not that of Bohr, also assumes that the concept of quantum objects, such as an electron or photon, is only applicable at the time of observation but not independently of observations [1,2,3]. This aspect of the present interpretation is, however, not discussed here. In any event, RWR interpretations place the ultimate character of the reality responsible for quantum phenomena beyond representation or, in the strong form of these interpretations such as that of Bohr or the one assumed here, even beyond conception. Hereafter, unless qualified, RWR interpretations refer to strong versions of them. A realist or ontological interpretation of quantum theory offers a representation or at least a conception of this reality, usually in terms of, or connected to, the mathematical formalism of QM or QFT (for a comprehensive discussion of RWR interpretations, see [1,2]).

Shortly before the paper containing his discovery of QM was published, Heisenberg wrote to Ralph Kronig: “What I really like in this scheme [QM] is that one can really reduce *all interactions* between atoms and the external world to transition probabilities” (Letter to R. Kronig, 5 June 1925; cited in [20] (v. 2, p. 242)). By referring to the “interactions between atoms and the external world”, this statement suggests that QM was only predicting the effects of these interactions observed in quantum phenomena. Quantum phenomena are defined in observational instruments and amenable, along with the observed parts of these instruments, to a treatment by classical physics. The later circumstance became crucial to Bohr’s interpretation, eventually leading him to his concept of the [quantum] phenomenon discussed below. Establishing these effects required special instruments. Human bodies are sufficient in some cases and are good models of the instruments used in classical physics. This is, however, not the case in quantum physics, which is irreducibly technological, or in relativity which is irreducibly technological as well because its effects cannot be perceived by human bodies alone. Their irreducibly technological nature, however, still allows relativity theory to be a classically causal, in fact deterministic, theory, as well as, indeed in the first place, a realist theory. This is because the observational instruments used (rods and clocks) allowed one to observe the systems considered without disturbing them and (ideally) mathematize their independent behavior. This is no longer possible in considering quantum phenomena. This fact poses major difficulties for realism, even if it does not preclude it. Heisenberg’s view just cited was adopted by Bohr as a defining feature of his interpretation in all its versions, with a special emphasis on the role of classical physics in describing quantum phenomena and data contained in them. It should be kept in mind that Bohr adjusted his views, sometimes significantly, a few times, eventually arriving at his ultimate RWR interpretation around 1937, after a decade of debate with Einstein. This requires one to specify to which version of his interpretation one refers, which I do as necessary while focusing on his ultimate interpretation, unavoidably in the present *interpretation* of his interpretation. Unless qualified, “Bohr’s interpretation” refers to his ultimate interpretation. The designation of “the Copenhagen interpretation” requires even more qualifications as concerns whose interpretation it is, say, that of Heisenberg, Dirac, or von Neumann, which compels me to avoid this designation entirely. 

The idea of placing the ultimate reality responsible for quantum phenomena beyond representation was adopted by Bohr in the immediate wake of Heisenberg’s discovery of QM (before Schrödinger’s wave mechanics was introduced):

In contrast to ordinary mechanics, the new quantum mechanics does not deal with a space–time description of the motion of atomic particles. It operates with manifolds of quantities which replace the harmonic oscillating components of the motion and symbolize the possibilities of transitions between stationary states in conformity with the correspondence principle. These quantities satisfy certain relations which take the place of the mechanical equations of motion and the quantization rules [of the old quantum theory] [14] (v. 1, p. 48).

Bohr’s strong form of the RWR interpretation, which placed the reality ultimately responsible for quantum phenomena beyond conception, was introduced a decade later, shaped by Bohr’s debate with Einstein. In his 1937 article, arguably introducing this interpretation, he referred to “our not being any longer in a position to speak of the autonomous behavior of a physical object, due to the unavoidable interaction between the object and the measuring instrument” which, by the same token, entails a “renunciation of the ideal of [classical] causality in atomic physics” [21] (p. 87). Obviously, if we could form a concept of this behavior, we would be able to say something about it. In reflecting on this interpretation in 1949, in response to Einstein’s criticism, Bohr said: “In quantum mechanics, we are not dealing with an arbitrary renunciation of a more detailed analysis of atomic phenomena, but with a recognition that such an analysis is *in principle* excluded’’ [14] (v. 2, p. 62). 

As noted, “the unavoidable interaction between the object and the measuring instrument” defined this new epistemological situation, always dealing, to return to Heisenberg’s formulation, with “the interactions between atoms and the external world” and “transition probabilities” between these interactions. As Bohr argued from the outset of his interpretation of QM, in classical physics and relativity theory “our… description of physical phenomena [is] based of the idea that the phenomena concerned may be observed *without disturbing them appreciably*”, which also enables one to identify these phenomena with the objects considered [14] (v. 1, p. 53; emphasis added). By contrast, “any observation of atomic phenomena will involve an *interaction [of the object under investigation] with the agency of observation* not to be neglected” [14] (v. 1, p. 54; emphasis added). This argument was retained in all versions of his interpretation, all grounded in the irreducible role of observations instruments as a necessary part of any agency of observation in the constitution of quantum phenomena. One should keep in mind the subtle nature of this contrast: the interaction between the object under investigation and the agency of observation *gives rise* to a quantum phenomenon, in fact in a unique act of creation, rather than *disturbs* it [14] (v. 2, p. 64). Relativity theory, again, represents a step in this direction insofar as, in contrast to Newtonian mechanics, space and time were no longer seen as preexisting (absolute) entities then measured by rods and clocks but were instead defined by the latter in each reference frame. Still, the interference of observational instruments into the behavior of the objects considered could be disregarded, thus allowing, as in classical physics, the identification of these objects with observed phenomena for all practical purposes. As a result, the objects under investigation could be considered independently of their interactions with measuring instruments.

Disregarding this interference is no longer possible in considering quantum phenomena empirically and hence even in realist interpretations of QM or in alternative theories such as Bohmian mechanics. This impossibility eventually led Bohr to his concept of “phenomenon” as applicable in quantum theory under the condition of an RWR interpretation, introduced by him at the same time (around 1937). Bohr adopted the term “phenomenon” to refer only to what is observed in measuring instruments:

I advocated the application of the word phenomenon exclusively to refer to *the observations obtained under specified circumstances*, including an account of the whole experimental arrangement. In such terminology, the observational problem is free of any special intricacy since, in actual experiments, all observations are expressed by unambiguous statements referring, for instance, to the registration of the point at which an electron arrives at a photographic plate. Moreover, speaking in such a way is just suited to emphasize that the appropriate physical interpretation of the symbolic quantum-mechanical formalism amounts only to predictions, of determinate or statistical character, pertaining to individual phenomena appearing under conditions defined by classical physical concepts [describing the observable parts of measuring instruments] [14] (v. 2, p. 64).

Technically, one can no longer speak of the electron having *arrived* at a photographic plate, which implies a classical-like motion, rather than that the spot on the plate registers, in the corresponding phenomenon, the electron as the considered quantum object. This is, however, clearly what Bohr means given what else he says in this article. The difference between phenomena and objects has its genealogy, in modern times (it had earlier precursors), in Kant’s distinction between objects as things-in-themselves in their independent existence and phenomena as representations created by our mind. As defined by strong RWR interpretations, however, Bohr’s or the present view is more radical than that of Kant. While Kant’s things-in-themselves are assumed to be beyond knowledge, they are not assumed to be beyond conception, at least a *hypothetical* conception, even if such a conception cannot be guaranteed to be correct and is only practically justified in its applications [22] (p. 115). By contrast, in (strong) RWR interpretations, what is practically justified is not any possible conception of the ultimate reality responsible for quantum phenomena, but the impossibility of such a conception.

Bohr came to see quantum phenomena as revealing “a novel feature of atomicity in the laws of nature”, “disclosed” by “Planck’s discovery of the quantum of action [*h*], supplementing in such unexpected manner the old [Democritean] doctrine of the limited divisibility of matter” [15] (p. 94). Atomicity and thus discreteness or discontinuity in quantum physics initially emerged on this Democritean model, beginning with Planck’s discovery of the quantum nature of radiation in 1900. This discovery led Planck to his concept of the quantum of action *h* physically defining this discontinuity and Einstein’s concept of a photon as a particle of light in 1906. The situation, however, especially following the discovery of QM, revealed itself to be more complex. This complexity led Bohr to his concepts of phenomenon and atomicity as part of his ultimate RWR interpretation.

Bohr’s concept of atomicity is essentially equivalent to that of phenomenon (every instance of “atomicity” is a phenomenon and vice versa) but highlights such features of quantum phenomena as their individual, even unique nature and their discreteness relative to each other as follows. First, Bohr’s concept of phenomena implies that nothing about quantum objects themselves could ever be extracted from phenomena. This impossibility defines what Bohr calls the wholeness or indivisibility of phenomena, which makes them “closed”: the ultimate constitution of reality that led to the emergence of any quantum phenomenon is sealed within this phenomenon and cannot be unsealed. As he says,

The essential wholeness of a proper quantum phenomenon finds indeed logical expression in the circumstance that any attempt at its well-defined subdivision would require a change in the experimental arrangement incompatible with the appearance of the phenomenon itself. …every atomic phenomenon is closed in the sense that its observation is based on registrations obtained by means of suitable amplification devices with irreversible functioning such as, for example, permanent marks on a photographic plate, caused by the penetration of electrons into the emulsion. …the quantum-mechanical formalism permits a well-defined application referring only to such closed phenomena. [14] (v. 2, pp. 72–73; also p. 51).

In this way, phenomena acquire the property of “atomicity” in the original Greek sense of an entity that is not divisible any further. This concept, however, now applies at the level of phenomena, rather than referring, along Democritean lines, to indivisible physical entities, “atoms”, of nature. Bohr’s scheme enables him to transfer to the level of observable configurations manifested in measuring instruments all the key features of quantum physics—discreteness, discontinuity, individuality, and atomicity (indivisibility)—previously associated with quantum objects themselves. As is Bohr’s concept of phenomenon, the concept of “atomicity” is defined in terms of individual effects of quantum objects on the classical world as opposed to Democritean atoms of matter itself. “Atomicity” in Bohr’s sense refers to physically complex and hence physically subdivisible entities, and no longer to single physical entities, whether quantum objects themselves or even point-like traces of physical events. In other words, these “atoms” are individual phenomena in Bohr’s sense rather than indivisible atomic quantum objects to which one can no longer ascribe atomic physical properties any more than any other properties. Any attempt to “open” or “cut through” a phenomenon (this would require a different experiment, and hence one is never really cutting through the same phenomenon, which confirms the uniqueness of each) can only produce yet another closed individual phenomenon, a different “atom” or set of such “atoms”, leaving quantum objects themselves inaccessibly inside phenomena.

Importantly, as defined by “*the observations* [already] *obtained under specified circumstances*”, phenomena refer to events that have already occurred and not to possible future events, such as those predicted by QM. This is the case even if these predictions are ideally exact, which they can be in certain circumstances, such as those of EPR-type experiments. The reason that such a prediction cannot define a quantum phenomenon is that a prediction for variable *Q* (for example, that related to a coordinate, *q*) cannot, in general, be assumed to be confirmed by a future measurement in the way it can be in classical physics or relativity theory, where are all possible measurable quantities are always defined simultaneously. In quantum physics, one can always, instead of the predicted measurement, perform a complementary measurement, that of *p* (the momentum), which leaves any value predicted by using *Q* entirely undetermined by the uncertainty relations. This measurement would in principle preclude associating a physical reality corresponding to a coordinate *q* when one measures *p* [1] (pp. 210–212). This is why classical causality does not apply in the way it does in classical physics, even when probability is used there or in relativity (which is a deterministic theory, in which all predictions are ideally exact). This point has major implication for understanding the EPR experiment and countering EPR’s arguments along the lines of Bohr’s reply [1] (pp. 227–257). I use capital vs. small letters to differentiate Hilbert-space operators, *Q* and *P*, associated with predicting the values of measured quantities, *q* and *p*, observed on measuring instruments. One can never speak of both variables unambiguously, even if they are associated with measuring instruments, while any references, even that to a single property of a quantum object, are not possible at all in RWR interpretations even at the time of observation, let alone independently. In classical physics, this difficulty does not arise because one can, in principle, always define both variables simultaneously and unambiguously speak of the reality associated with both and assign them to the object itself. By contrast, in a quantum experiment one always deals with a system containing an object and an instrument. Thus, in considering quantum phenomena (strictly defined by observation), there is, on the one hand, always discrimination between an object and an instrument and, on the other, the impossibility of separating them. This impossibility compelled Bohr to speak of “the essential ambiguity involved in a reference to physical attributes of objects when dealing with phenomena where no sharp distinction can be made between the behavior of the objects themselves and their interaction with the measuring instruments” [14] (v. 2, p. 61). By contrast, a reference to what is observed can, as classical, be unambiguous and communicated as such.

This interpretation radically changes the meaning of all elements and relations between them in Diagram A in the case of QM or QFT, in accord with Bohr’s statement with which I started, modifying it to fit all physics, which is, however, in accord with Bohr’s view. To cite Bohr’s actual statement defining this diagram in quantum theory,

The essential lesson of the analysis of measurements in quantum theory is thus the emphasis on the necessity, in the account of the phenomena, of taking the whole experimental arrangement into consideration, in complete conformity with the fact that all unambiguous interpretation of the quantum mechanical formalism involves the fixation of the external conditions, defining the initial state of the atomic system concerned and the character of the possible predictions as regards subsequent observable properties of that system. Any measurement in quantum theory can in fact only refer either to a fixation of the initial state or to the test of such predictions, and it is first the combination of measurements of both kinds which constitutes a well-defined phenomenon. [15] (p. 101).

Technically, in Bohr’s definition of the concept, one deals here with two phenomena corresponding to two events in Diagram A. This statement need not mean that Bohr’s concept of phenomenon applies to two measurements or, and in particular, that it can refer to a prediction, which is why Bohr’s speaks of “the test of … predictions”, that is, already performed experiments. The point is that one must specify two measurements and two instruments prepared: the first is the initial, actual measurement or phenomenon and the second is a possible future measurement or phenomenon that would enable us to verify our probabilistic prediction or our statistical predictions after repeating the experiments many times. As Bohr said in the same article, “It is certainly far more in accordance with the structure and interpretation of the quantum mechanical symbolism, as well as with elementary epistemological principles, to reserve the word phenomenon for the comprehension of the effects observed under given experimental conditions” [15] (p. 105). Thus, as his other statements confirm as well, a phenomenon is defined by an already performed measurement as an effect of the interactions between quantum objects and the apparatus but never by a prediction. Then, the first description above is contextualized as referring to “that all unambiguous interpretation of the quantum mechanical formalism involves the fixation of the external conditions, defining the initial state of the atomic system concerned and the character of the possible predictions as regards subsequent observable properties of that system”. The second refers to the test of any such prediction. One needs both arrangements and both phenomena (defined when both measurements are performed) to test our predictions in quantum physics in repeated experiments because our predictions are in general probabilistic or statistical.

Accordingly, observational instruments must now be added to Diagram A.

E_0_ (q_0_)               E_1_ (q_1_) t_0_                   t_1_ Instrument 1           Instrument 2

The diagram now reflects the fundamental difference between classical physics or relativity and quantum. In classical physics or relativity, the interference of measuring instruments could, at least in principle, be neglected or controlled, allowing one dealing with individual systems to identify the observed phenomenon with the object considered. In quantum physics, this interference cannot be controlled and hence cannot be neglected, which makes the phenomena observed in measuring instruments, the only observable phenomena, different from quantum objects considered. As stated, nobody has ever seen an actual electron or photon or any quantum object, as such. One can only observe traces of their interactions with measuring instruments. I indicate this in the diagram leaving the space between E_0_ (q_0_) and E_1_ (q_1_) empty, as Bohr often does in his diagrams illustrating quantum experiments (e.g., [14] (v. 2, pp. 48–55)). In dealing with quantum phenomena, deterministic predictions are not possible even in considering the most elementary quantum systems. The repetition of identically prepared quantum experiments in general leads to different recorded data, and unlike in classical physics, this difference cannot be diminished beyond the limit defined by *h* by improving the capacity of our measuring instruments. These data are both those recorded of the initial measurement E_0_ enabling a prediction and those of the second measurement E_1_ which would verify this prediction. These recordings are in general different either when one repeats the whole procedure in the same set of experimental arrangements or when one builds a copy of the apparatus and sets it up in the same way in order to verify experimental findings by others. This makes the verification in quantum physics in general statistical.

In RWR interpretation, QM or QFT only predict the effects of these interactions observed in the instruments used, without representing how these effects come about. This procedure replaces measurement in the classical sense (of measuring preexisting properties of objects) with the establishment of each quantum phenomenon as each time a unique act of creation. Observed phenomena can be treated classically, without measuring the properties of quantum objects, at least in an RWR interpretation. In fact, RWR interpretation responds to this experimental situation, arising, in these interpretations, because of the irreducible role of measuring instruments in the constitution of quantum phenomena. Returning to Diagram A, in its quantum version, in RWR interpretations, no representation or even conception of how the transition between E_0_ (q_0_) and E_1_ (q_1_) happens is possible; but it is possible to find transition probabilities between these events. To do so, one needs a theory such as QM or, in high-energy regimes, QFT, which predicts these probabilities or statistics. (The difference between probability and statistics is put aside, although it may affect an interpretation, including an RWR one, of QM or QFT.) Quantum phenomena and the data observed there are, in Bohr or the present view, represented by classical physics.

One can, for example, use Schrödinger’s equation (considering it in one dimension for simplicity),

$$i\hbar \frac{\partial \psi (x,t)}{\partial t}=\left[-\frac{\hbar^{2}}{2m}\frac{\partial }{\partial x^{2}}+V(x,t)\right]\psi \left(x,t\right),$$

for making a prediction concerning a future coordinate measurement associated with an electron on the basis of one previously performed at time *t_0_* and position measurement *q_o_*. This already performed measurement enacts the transition from the classical reality observation to the ultimate “quantum” reality responsible for the observed quantum phenomena. One cannot associate any concept of “quantum” with the ultimate reality responsible for quantum phenomena in RWR interpretations, because one cannot associate any concept with this reality at all. I speak of this reality as “quantum” only because classical physics, which describes the data classically observed in two experiments considered, cannot predict them. In this (one-dimensional) case, the wave function ψ(x,t) assigns a complex number to each point *x* at each moment in time *t*, *m* is the mass of the object considered, and V(x,t) is a potential that represents the domain of the ultimate reality responsible for the phenomena observed. By Born’s rule, the wave function is associated with the probability amplitude by providing the probability density by the square modulus of |*ψ*〉, ‖*ψ*‖^2^, which is always positive and can be normalized so the probability is between zero and one. Then, to confirm such a prediction, one sets up a suitable observational device and makes a new observation at time *t*_1_ registering outcome *q*_1_ of the experiment, thus predicted. The instrument needs to be prepared in accordance with this prediction for the coordinate measurement. This is because one can always perform a different type of measurement, that of the momentum, *p*, at *t*_1_, which irrevocably disables verifying the prediction concerning *q*. This is the transition from the “quantum” to the classical in this experiment (the above qualification concerning the term “quantum” still applies).

As Bohr emphasized, in spite or even because of their radical epistemological feature his and, by implication, all RWR interpretations remain fully consistent with “the basic principles of science” [23] (p. 697). That includes “the unambiguous logical representation of relations between experiences” and hence the possibility of unambiguously communicating the contents of these experiences [14] (v. 2, p. 68). Thus, his concept of complementarity (explained below) was expressly introduced to ensure the possibility of maintaining these principles in quantum physics under an RWR interpretation [23] (p. 699). Indeed, one of Bohr’s aims was to argue that the basic principles of science are not threatened by the radical epistemological implications of quantum theory, especially in RWR interpretations, as many worried at the time and still do. Of course, what constitutes the basic principles of science may be viewed differently, and it could be and has been debated. There is a nearly unanimous agreement on the logical consistency and unambiguity of communication in mathematics and physics or in physics on the consistency of physical theories with the experimental evidence available and their capacity, in modern physics by means of mathematics, for predicting the outcomes of the experiments considered by them. (In general, a physical theory must be consistent with all available experimental evidence or confirmed physical laws rather than only those within its purview). For Bohr, to be in accord with these requirements as quantum theory was, it was sufficient to see quantum theory as conforming to “the basic principles of science”. The present view is the same. On the other hand, many physicists and philosophers of science, Einstein famously among them, saw a representation of how physical phenomena, quantum phenomena included, come about, in other words, realism, as an equally basic principle of science. 

It is not coincidental that in stating that “when speaking of conceptual framework, we refer merely to the unambiguous logical representation of relations between *experiences*”, Bohr refers to experiences rather than experiments as one might expect in dealing with physics [14] (v. 2, p. 68; emphasis added). The reason is that Bohr makes a broader point. Our experiences in science, or mathematics, combine individual aspects with shared ones, conforming to the unambiguity communication. “Individual” and “shared” are arguably better terms than “subjective” and “objective”, although this depends on how one defines subjectivity and objectivity. Science and especially mathematics must maximally reduce the ambiguity of defining and communicating their findings. This was the definition of “objectivity” in Bohr vs. realism in which “objectivity” also and primarily refers to the “objective” representation of the ultimate reality responsible for quantum phenomena. This type of representation was “*in principle* excluded” in Bohr’s RWR interpretation [14] (v. 2, p. 62). A representation by means of classical physics is possible, and in Bohr or the present interpretation assumed necessary in considering what is observed as quantum phenomena. (It is possible to have an RWR interpretation apart from this assumption.) 

The uncertainty relations exemplify this situation. They are a defining feature of quantum phenomena rather than of QM, the predictions of which are, however, fully in accord with the uncertainty relations. This qualification is important. While this is a major testimony to the accord between QM and the experimental data it considers, the uncertainty relations are an experimentally established fact, a law of nature, independent of any theory, and could in principle be predicted by an alternative theory. (Bohmian mechanics predicts them.) An uncertainty relation is represented by formula Δ*q*Δ*p* ≅ *h*, where *q* is the coordinate, *p* is the momentum in the corresponding direction, which are *measurable quantities* observed in instruments, *h* is the Planck constant, and Δ is the standard deviation, which measures the amount of variation of a set value. There is nothing unambiguous about the formula itself. It represents an experimentally confirmed law independent of any theory, although a physical interpretation of the uncertainty relations is subtle. It suffices to note here that the uncertainty relations are not a manifestation of the limited accuracy of measuring instruments, because they would be valid even if we had perfect measuring instruments. In classical and quantum physics alike, one can only measure or predict each variable within the capacity of our measuring instruments. In classical physics, however, one can in principle measure both variables simultaneously within the same experimental arrangement and improve the accuracy of this measurement by improving the capacity of our measuring instruments, in principle indefinitely. The uncertainty relations prevent us from doing so for both variables in quantum physics, regardless of this capacity. Accordingly, what the uncertainty relations state, and state *unambiguously*, is that the simultaneous exact measurement or prediction of both variables (always possible, at least ideally and in principle, in classical physics) is not possible. On the other hand, it is always possible to measure or predict each variable ideally exactly. It is also possible to measure both inexactly. For Bohr, however, and following him here, the uncertainty relations mean that one cannot unambiguously consider or even define both variables simultaneously but can only define one at each moment in time and thus make unambiguous statements about it.

This situation is understood by Bohr in terms of complementarity, his most famous concept. It is defined by the mutual exclusivity of certain measurements or predictions at any given point in time and yet the possibility, by a conscious decision, of measuring either one or the other exactly at any given point in time, which, however, in principle preclude doing the same for the other, complementary variable. The role of this conscious decision is crucial in quantum physics and is one of the key differences between it and classical physics or relativity theory, which do not contain either the uncertainty relations or complementarity [1] (pp. 207–218). By the same token, in accord with quantum causality as probabilistic causality, one can only make, by means of QM, the corresponding prediction, which is in general probabilistic, concerning a future measurement of one variable while precluding one from doing so for the other [1] (pp. 207–218). Complementarity thus helps to establish the possibility of unambiguous definition and communication of everything involved in considering quantum phenomena, including, again, the uncertainty relations.

The mathematical formalism of QM or QFT can also be unambiguously communicated, as can all mathematics used in quantum theory or any mathematics as such. Indeed, after defining in the passage cited above “a conceptual framework” as “the unambiguous logical representation of relations between experiences”, Bohr expressly comments on mathematics and its “special role in physics:”

A special role in physics is played by mathematics which has contributed so decisively to the development of logical thinking, and which by its well-defined abstractions offers invaluable help in expressing harmonious relationships. Still, in our discussion… we shall not consider pure mathematics as [an entirely] separate branch of knowledge, but rather a refinement of general language [and concepts], supplementing it with appropriate tools to represent relations for which ordinary verbal expression is imprecise and cumbersome. In this connection, it may be stressed that, just by avoiding the reference to conscious subject which infiltrates daily language, the use of mathematical symbols secures the unambiguity of definition required for objective description [14] (v. 2, p. 68; emphasis added).

I add concepts because it is difficult to dissociate words from concepts. One exception of a word without a concept, if one can still speak of a word in this case, is the word reality in “reality without realism”. As beyond conception, either word is designed not to have a concept or signified associated with it. At the same time, reality without realism is a philosophical concept, a concept that refers to the impossibility of a concept or signified associated with the word reality or ideality. A reality without realism is, however, always part of the concept defined in the standard way from which such a reality or ideality is inferred, such as in quantum physics the concept of a quantum phenomenon observed in measuring instruments. Quantum phenomena are described by ordinary language supplemented by mathematics, in this case that of classical physics. Refinement used by Bohr is a term suggesting a capacious concept, likely also referring to a highly refined nature of the abstract mathematics used in QM and QFT or related contemporary mathematics. Bohr’s brother, Harald Bohr, with whom Bohr talked daily, was a world-renowned mathematician working in functional analysis and related fields and who was the founder of the field of almost periodic functions.

It follows that “intersubjectivity”, a much debated to topic in recent discussion, is automatic under the conditions just outlined (defining both the present and Bohr’s ultimate interpretation), beginning with the fact that objectivity is defined here as the possibility of unambiguous communication. Mathematics and science capitalize on this possibility. This is also possible in experimental science in communicating the outcomes of experiments by means of general language, accompanied by technical terminology. Hence, as Bohr said above, “all *unambiguous* interpretation of the quantum mechanical formalism involves the fixation of the external conditions, defining the initial state of the atomic system concerned and the character of the possible predictions as regards subsequent observable properties of that system”. On the other hand, objectivity in the (realist) sense of representing the “object” considered, material in science or mental in mathematics, is a different matter. This is because of a possible application of an RWR interpretation, which precludes any such representation while allowing for objectivity in the sense of unambiguous communication. This kind of objectivity may be assumed at least for practical purposes in classical physics or relativity. But it cannot be assumed, even for practical purposes, in considering the ultimate reality responsible for quantum phenomena in RWR interpretations, which place this reality beyond representation or even conception. It follows, then, that

(1) the mathematical formalism of QM or QFT is unambiguously communicable;
(2) the observation or measurement is classical (cum special relativity in high-energy regimens) and hence is unambiguously communicable.

Objectivity as unambiguous communication ⟹ intersubjectivity,

but

(3) the reality responsible for quantum phenomena is beyond conception, and thus cannot be involved in any communication or even be known to or be conceivable by any human subject.

This reality is thus discontinuous relative to any conception, physical, philosophical, or mathematical, that we can form. On the other hand, our conceptions may enable us to connect, in terms of probabilistic predictions, possibly by means of *continuous* mathematics, to quantum phenomena, phenomena that are always discrete, and hence physically *discontinuous*, relative to each other. This situation defines quantum physics as “the physics of a discontinuum”, to adopt the term of Heisenberg, Max Born, and Pasqual Jordan in their paper finalizing matrix mechanics along similar lines [24].

## 4. Quantum Continuum: Mathematics Before Physics

I argue that while the difference between quantum and classical (or relativistic) phenomena is defined by new physical features (codified by Planck’s constant *h*), the difference between quantum and classical theory is not defined by a mathematical representation of these features. This difference is defined by a new mathematical, “abstract” scheme established on the basis of purely mathematical postulates, a scheme that relates, in general probabilistically, to quantum phenomena as defined by these new physical features or postulates. We are accordingly fortunate that our mathematics allows us to develop the formalism that correctly predicts the outcomes of quantum experiments, admittedly only probabilistically, which, however, is strictly in accord with what is experimentally possible, at least as things stand now. According to Heisenberg,

It is not surprising that our [ordinary] language [and concepts] should be incapable of describing processes occurring within atoms, for… it was invented to describe the experiences of daily life, and these consist only of processes involving exceedingly large numbers of atoms. …Fortunately, *mathematics is not subject to this limitation*, and it has been possible *to invent a mathematical scheme*—the quantum theory [QM]—which seems entirely adequate for the treatment of atomic processes [25] (p. 11; emphasis added).

It is important that Heisenberg refers to “*processes* involving exceedingly large numbers of atoms”. Quantum objects, such as Bose–Einstein condensates or Josephson’s devices, could be macroscopic, but their quantum nature is still defined by their microscopic quantum constitution and thus subatomic quantum processes. This constitution precludes their description by means of ordinary language or concepts, and in RWR interpretations, by any means. Heisenberg’s main point amounts to the fact that our language and thought are the product of our evolutionary biological and specifically neurological development defined by the experiences consisting only of processes dealing with huge numbers of atoms. Accordingly, there is no special reason to assume that they are able to describe nature on the atomic or still smaller scales, or conversely on very large, cosmological scales. One might instead doubt that one can do so even mathematically, as Heisenberg eventually assumed (e.g., [26] (pp. 59–75, 145, 167–186)) given that mathematics, too, is only human and as such is the product of the same evolutionary development. We are fortunate to be capable of using it to predict quantum phenomena. Mathematics, however, especially *abstract* mathematics, such as that used in QM and QFT, does have a great, even if not complete, degree of freedom from the limitations of daily language and thinking. This freedom also ensures the unambiguity of mathematical or mathematical–physical communication within much broader limits, as Bohr noted in his comment, cited earlier, to the effect that “by avoiding the reference to conscious subject which infiltrates daily language, the use of mathematical symbols secures the unambiguity of definition [and communication] required for objective description” [14] (v. 2, p. 68). Modern mathematics and physics based on it ultimately break with ordinary language or thinking more radically. In physics, this break became pronounced in relativity theory and then quantum theory, where it became complete in considering the ultimate reality responsible for quantum phenomena, at least in RWR interpretations, beginning with that of Heisenberg at the time of his invention of quantum mechanics in 1925. (Later on, Heisenberg adopted a more realist view of quantum theory.) In reflecting on this situation in his later book, Heisenberg said:

There is no description of what *happens* to the system between the initial observation and the next measurement. …The demand to “describe what happens” in the quantum-theoretical process between two successive observations is a contradiction in adjecto, since the word “describe” refers to the use of classical concepts, while these concepts cannot be applied in the space between the observations; they can only be applied at the points of observation. … [T]he problems of language are really serious. We wish to speak in some way about the structure of the atoms [as made of particles] and not only about ‘facts’—the latter being, for instance, the black spots on a photographic plate or the water droplets in a cloud chamber. However, we cannot speak about the atoms in ordinary language [26] (pp. 57, 145, 178–179).

Nor is it possible in terms of ordinary concepts from which ordinary language is indissociable. Heisenberg’s formulation does allow for a *mathematical representation* of the reality between observations, defining quantum phenomena, which was indeed a view adopted by Heisenberg at the time of this statement [26] (pp. 59–75, 145, 167–186) [1] (pp. 75–76). The words “happens” or even “physical” (or any word) need, accordingly, no longer be part of this representation, which only requires mathematical symbols, perhaps defined with the help of ordinary language. Heisenberg saw this mathematical representation in terms of symmetry groups and defined elementary particles accordingly, as the irreducible representations of such groups. (These representations are designated below as “Galois atoms”, without considering these “atoms” as particles in a physical sense.) Heisenberg still assumed, however, the existence of a physical reality responsible for this situation, thus also maintaining affinities with Bohr’s view. According to Bohr’s view, however, or to the present view, this reality is an RWR-type reality and hence, as beyond all conception, is also beyond any mathematical conception, let alone mathematical representation. Heisenberg also assumed, again, in accord with Bohr’s view, that what is observed in measuring instruments as quantum phenomena is described classically, which also mandates the arrow of time, because the sequence of our experiments is irreversible [1] (pp. 207–218).

It remains the case, however, that mathematics, especially *abstract* mathematics, such as that used in QM and QFT, has a great, even if not unlimited, freedom from the limitations of daily language and thinking. As such, it enables us to *relate* to things in nature and (in mathematics) mind which are, as forms of RWR-type reality, beyond the reach of thinking, including mathematical thinking. In QM and QFT it does so by enabling us to estimate probabilities or statistics of quantum events. In fact, it is equally fortunate that nature allows us to use such a “mathematical scheme”, because the freedom of mathematics from the limitations of common language and concepts does not a priori guarantee that any such scheme will work, and it might not work beyond the scales now handled by QFT. 

In his invention of QM, Heisenberg took advantage of this freedom of mathematics, especially abstract mathematics, in this case matrix algebra, from the limitations of language. He also found this new way to connect abstract mathematics to physics in his discovery of QM. This made new physics engendered by abstract mathematical schemes, either invented, as they were by Heisenberg (technically reinvented, because his matrix variables already existed in mathematics), or borrowed from already available mathematics. This approach was quickly extended by Dirac to QED and thus physics in high-energy quantum regimes. Dirac expressly advocated this “method of advance”:

The most powerful method of advance that can be suggested at present is to employ all the resources of pure mathematics in attempts to perfect and generalise the mathematical formalism that forms the existing basis of theoretical physics, and after each success in this direction, to try to interpret the new mathematical features in terms of physical entities [27] (p. 1).

While, on this occasion, Dirac saw this “method” as the best way of possibly dealing with the infinities of QED (leading to renormalization), he already used the same method in his work on QM and on QED, especially in deriving Dirac’s equation. So did Heisenberg in creating QM in 1925, and Heisenberg’s original paper served as an inspiration for Dirac. In this method, one moves, *advances* from mathematics, *abstract mathematics*, to physics, rather than from physics to mathematics representing this physics. 

As I argue here, in all modern, mathematical–experimental physics, with mathematics again coming first in this conjunction, the creation of new physics is a creation of new mathematics for physics, possibly by adopting some previously existing mathematics. In the view, adopted by Heisenberg and Dirac at the time, QM was no longer defined, as was classical physics or relativity theory, by the aim of representing the ultimate nature of the reality responsible for quantum phenomena. At the very least, Dirac and Heisenberg were not concerned with this task, assumed to be difficult or even hopeless by them. In this, they, Heisenberg expressly, followed Bohr’s abandoning the mechanical description of quantum jumps as a “hopeless” task in his 1913 atomic theory [28] (p. 7) [29]. Instead, their theories aimed at using the mathematical formalism of quantum theory for probabilistic predictions of the data registered in quantum phenomena and only such predictions. No other predictions are, again, possible on experimental grounds. By the same token, while the main differences from previous physics were defined by new physical features of quantum phenomena, the main differences between quantum theory and classical physics or relativity theory were defined by a new mathematical, “abstract” scheme defined by purely mathematical postulates. This scheme only relates, in general probabilistically, to quantum phenomena defined physically. RWR interpretations respond to and ground this situation by precluding representing by any means, including mathematical ones, how quantum phenomena and thus their special, “quantum” features come about. Quantum phenomena were assumed by Heisenberg to be represented by classical physics rather than by QM. This assumption was often abandoned subsequently, especially following von Neumann’s formalism, and is not technically required in an RWR interpretation. It was, however, made by Heisenberg, who also continued to maintain it later, when he shifted his view of quantum theory to a form of mathematical realism. Thus, as against classical physics, quantum theory requires two separate physical theories. (This, as noted, is also true in relativity theory, where measuring instruments—rods and clocks—are also described classically, while, however, maintaining a realist representation at all levels). I considered Heisenberg’s derivation of QM in his first paper in detail previously (most recently in [1] (pp. 101–127)). Accordingly, rather than rehearsing his derivation here, I sum up the key implications of this derivation for my argument in this article. 

First of all, Heisenberg expressly used mathematics never previously used in physics, that of unbounded infinite-dimensional matrices over C. He was famously unaware of the existence of matrix algebra and reinvented it, although unbounded infinite matrices were not previously studied. The noncommutative nature of his variables was one of the unexpected and implicative new features of Heisenberg’s algebra, with, say, *PQ* − *QP ≠ 0* for the corresponding variables, associated, respectively, with momentum *p* and coordinate *q* (Heisenberg only considered *q* in the toy model of his first paper), which were probabilistically predictable as actual measurable physical quantities using his mathematics. They were also supplemented by additional rules, eventually absorbed by Born’s rule, which allowed one to move from complex to real quantities necessary for defining the probabilities in question. These variables (replacing the functions of the real variables of classical mechanics) conceived by Heisenberg in terms of linear algebra as matrix elements were eventually defined more formally as Hermitian operators in Hilbert spaces over C. 

All the key features of the formalism were contained in Heisenberg’s paper, apart from Schrödinger’s equation. It was introduced by Schrödinger a few months later. The main parallel mathematical innovation (when time was considered) was the complex wave function, $\psi \left(t\right)$, a vector in an infinite-dimensional complex Hilbert space, which enables one to predict the probabilities of quantum events. Indeed, Schrödinger initially intended his wave function to represent, in wave terms, moreover deterministically, the ultimate reality responsible for quantum phenomena. His hope of achieving this goal became more difficult and was abandoned by him soon after he introduced his time-dependent equation. This equation required a complex wave function, initially defined by him as a real entity for his time-independent equation, and made its predictions fundamentally probabilistic, just as they were in Heisenberg’s scheme all along [1] (pp. 145–166). As stated, for one dimension, Schrödinger’s time-dependent equation is

$$i\hbar \frac{\partial \psi (x,t)}{\partial t}=\left[-\frac{\hbar^{2}}{2m}\frac{\partial }{\partial x^{2}}+V(x,t)\right]\psi \left(x,t\right).$$

QM was quickly recast into Hilbert-space form (over C) by von Neumann, who also provided the first rigorous proof of the mathematical equivalence of Heisenberg’s and Schrödinger’s version, although this equivalence was by then widely assumed and used. Von Neumann eventually presented this version in his classic book [12], in part responding to yet another version of the formalism introduced by Dirac using his delta function [30]. The delta function was not considered a legitimate mathematical object at the time because its value is zero everywhere except at zero while its integral over the entire real line is equal to one, which is not a mathematically legitimate *function*. Von Neumann’s Hilbert space reformulation avoids it. The delta function was given mathematical legitimacy by means of a new concept, a distribution (in effect a functional), due to Sergei Sobolev and Laurent Schwarz (who introduced the term). This is an example of both the influence of physics on mathematics and the fact that one needs to give such ideas a mathematical rigor to make them part of mathematics. (As noted earlier, there are alternative formalisms, such as in terms of C* algebras or more recently monoidal categories, all thus far mathematically equivalent to the Hilbert space formalism, still used most). Von Neumann added Schrödinger’s equation as a postulate in his axiomatization. It was never strictly derived mathematically from simpler assumptions, but rather from a classical wave equation by ad hoc manipulations, in Schrödinger’s case “educated guesses”, to fit the experimental data considered [1] (pp. 145–166). 

Heisenberg’s thinking leading him to this discovery revolutionized the very practice of fundamental physics, expressly that of theoretical physics and, in effect, that of experimental physics when dealing with quantum phenomena. Bringing together the two main meanings of the word “experiment” (as a test and as innovative creation), the practice of quantum physics became the first practice of physics or science that is both, and jointly, *generatively* experimental and *generatively* mathematical. 

It is *generatively experimental* as experimental physics in its conventional sense because it is no longer defined, as in classical or relativistic physics, by tracking the independent behavior of the systems considered and measuring their independent properties. It is defined by *unavoidably* creating configurations of experimental technology containing traces of its interactions with nature that reflect the fact that what happens is unavoidably defined by what kinds of experiments we perform, by how we affect physical reality by a unique act of observation as creation. I qualify by “unavoidably” because, while the phenomena observed in classical physics or relativity may be affected by experimental technology and while we stage experiments there, in principle, one can observe these phenomena without appreciably affecting what is thus observed, which also allows one to see these phenomena as ideally representing the corresponding objects. Thus, one still follows what happens in any event. In quantum physics, the experiment is a creation of a new material and phenomenal configuration. This configuration enables one, by performing a measurement on the observable part of the instruments used, to define the probabilities for outcomes of future experiments, without it being possible to follow and track how these outcomes come about. In RWR interpretations, no assumption of the continuous (or any other, including discrete) concept of the ultimate nature of reality that gives rise to these events is possible. 

The practice of theoretical physics is *generatively mathematical* because it no longer consists, as in classical physics or relativity, in developing an idealized mathematical representation of quantum objects and behavior. It consists in inventing abstract mathematical schemes enabling us to predict, probabilistically or statistically, the outcomes of quantum events. These schemes are unrelated to and thus are not helped by our general phenomenal intuition, as are those of classical mechanics or, in a more limited way, relativity. On the other hand, they are not limited by this intuition either. Indeed, one experiments with mathematics as well. It is experimenting with abstract mathematics, divorced from our phenomenal intuition or even physical concepts, that, with Heisenberg, became the way to new theories in fundamental physics. Dirac’s work in QED was an even more striking example of this new approach to theoretical physics, which, as noted, he expressly advocated as “the most powerful method of advance that can be suggested at present” [27] (p. 1). Heisenberg was, however, the first practitioner of this method. This method is strictly in accord with my argument that the difference between quantum theory and classical physics (or relativity theory) is defined by purely mathematical postulates, so that quantum theory only relates, probabilistically, to the data-observed quantum phenomena, and is not connected to the physics responsible for the emergence of these phenomena.

I now consider, from this perspective, quantum entanglement, which is a unique feature of quantum physics that has been central to the last half-a-century discussion of quantum foundation. Schrödinger, who introduced the concept, saw it as a uniquely characteristic trait, *the* trait rather than merely one trait, of QM vs. classical physics or “classical lines of thought” in developing a physical theory:

When two systems, of which we know the states by their respective representatives, enter into temporary physical interaction due to known forces between them, and when after a time of mutual influence the systems separate again, then they can no longer be described in the same way as before, viz. by endowing each of them with a representative of its own. I would not call that *one* but rather *the* characteristic trait of quantum mechanics, the one that enforces its entire departure from classical lines of thought. By the interaction the two representatives have become entangled. Another way of expressing the peculiar situation is: the best possible knowledge of a *whole* does not necessarily include the best possible knowledge of all its *parts*, even though they may be entirely separate and therefore virtually capable of being ‘best possibly known,’ i.e., of possessing, each of them, a representative of its own. The lack of knowledge is by no means due to the interaction being insufficiently known—at least not in the way that it could possibly be known more completely—it is due to the interaction itself [31] (p. 555).

Quantum *phenomena* are never entangled because any observed quantum phenomenon arising from the situation defined and predicted by using the mathematics of entanglement separate, “disentangle” the object considered. Even speaking of entangled quantum objects requires caution because the combined entangled system (as a whole) precludes a sufficient knowledge to define each object [32] (p. 161). The “entangled” reality between quantum observations is unobservable and in RWR interpretations is beyond representation or even conception, including any concept, physical or mathematical, that one can associate with the term entanglement. There is no point speaking of parts and the whole between observations, and claims concerning it are unfalsifiable. In fact, one cannot speak of this unobservable and, in this interpretation, unrepresentable or even unthinkable reality itself as “entangled”. The knowledge in question is probabilistic, in accord with the nonadditive nature of probabilities of these predictions. This makes entanglement a feature of QM, enabling certain specific predictions (based on the data observed in certain specific previous experiments), by means of the mathematics of entanglement concerning the data appearing when the corresponding experiments, such as those dealing with quantum correlations, are performed. In short, it is not a feature of quantum phenomena, although it does predict certain features, such as correlations, pertaining to quantum phenomena. There are, of course, multiple interpretations of quantum entanglement, nearly as numerous as those of QM. For an instructive realist view, see [33].

In closing this section, I consider an axiomatically defined structure of quantum theory as a probabilistic theory, P_Q_, different from equally formally defined classical probabilistic theory, P_C_, by G. M. D’Ariano and co-workers in several publications, most extensively [34] (here, I follow [35]). This project offers an instructive illustration of my argument, or at least is open to this argument. I am not claiming that these authors necessarily subscribe to this argument.

A P_Q_ need not be QM (either finite or infinite dimensional), and classical theory need not be any probabilistic theory in classical physics. Accordingly, either a form of P_Q_ or P_C_ may remain in place for the phenomena considered, even if either QM or classical physics are replaced by some other quantum or classical theory thus defined. P_Q_ and P_C_ only differ by a single postulate, P6_Q_ “the purification postulate” (which accords with Schrödinger’s entanglement holism) vs. P6_C_ “the perfect joint discrimination postulate”. They share five other postulates.

P1 *Causality*: The probability of preparation is independent of the choice of observation.

P2 *Perfect discriminability*: Every state on the boundary of the convex set of states can be perfectly distinguished from some other state.

P3 *Local discriminability*: It is possible to discriminate any pair of states of composite systems using only local observations.

P4 *Compressibility*: For all states that are not completely mixed, there exists an ideal compression scheme.

P5 *Atomicity of composition*: The composition of two atomic transformations is atomic.

The sixth postulate is different for P_Q_ and P_C_.

P6_Q_ *Purification*: Every state has a purification. For a fixed purifying system, every two purifications of the same state are connected by a reversible transformation on the purifying system.

P6_C_ *Perfect joint discrimination: For any system, all pure states can be perfectly discriminated jointly.* Notice that P6_C_ forces P_C_ to restrict P_Q_’s pure states to a maximal set of perfectly discriminable ones. The purification postulate is defined as follows:

For every system *A* and for every pure state ρ∈St(A), there exists a system *B* and a pure state Ψ∈PurSt(AB), such that

(1) $$(\rho |\to A={\left(\Psi \right|}_{B\to (e)}^{A}$$

If two pure states Ψ and Ψ’ satisfy,

$${\left(\Psi ’\right|}_{B\to (e)}^{A}={\left(\Psi \right|}_{B\to (e)}^{A}$$

then there exists a reversible transformation *U*, acting only on system B, such that:

(2) $${\left(\Psi ’\right|}_{B}^{A}={\left(\Psi ’\right|}_{B\to [U]\to B}^{A}$$

In D’Ariano’s words, “We call Ψ a purification of ρ, with B [as a] purifying system. Informally, Equation (1) guarantees that we can always find a pure state of AB that is compatible with our limited knowledge of A alone. Furthermore, Equation (2) specifies that all the states of AB that are compatible with our knowledge of A are essentially the same, up to a reversible transformation on B. We call this property uniqueness of purification” [35] (p. 2).

Thus, in correspondence with my argument, while P1–P5 are essentially physical, P_Q_6 defining P_Q_ vs. P_C_ is *purely mathematical*. It can be, and here it is, expressed in part in ordinary language, which, in this case, unambiguously represents a mathematical concept. The main point, however, is that P_Q_6 reflects a purely mathematical nature of quantum theory, which only connects to the physics of quantum phenomena probabilistically, and hence in turn mathematically, without any connection to the physical emergence of these phenomena, in the way classical physics and relativity theory work. New mathematics drives new physics, which was the essence of Heisenberg’s and then Dirac’s “method” described by Dirac as “the most powerful method of advance” in quantum theory at the time. It may still be.

## 5. Continuity and Discontinuity, and Geometry and Algebra in Quantum Theory

My starting point in this section is Einstein’s assessment of Heisenberg’s “method” as essentially algebraic, in conjunction with one of his more positive assessments of QM, if still critical. In fact, it was more about an “element of truth” that it “has seized hold of” rather than QM itself. He said:

There is no doubt that quantum mechanics has seized hold of a beautiful element of truth and that it will be a touchstone for a future theoretical basis in that it must be deducible as a limiting case from that basis, just as electrostatics is deducible from the Maxwell equations of the electromagnetic field or as thermodynamics is deducible from statistical mechanics. I do not believe that quantum mechanics will be the starting point in the search for this basis, just as one cannot arrive at the foundations of mechanics from thermodynamics or statistical mechanics.

[P]erhaps the success of the Heisenberg method points to a purely algebraic method of description of nature, that is, to the elimination of continuous functions from physics. Then, however, we must give up, in principle, the space–time continuum [at the ultimate level of reality]. It is not unimaginable that human ingenuity will some day find methods which will make it possible to proceed along such a path. At present however, such a program looks like an attempt to breathe in empty space [36] (pp. 361, 378).

For Einstein, the proper basis for his “search for a more complete conception” of quantum phenomena had to be elsewhere [36] (pp. 361, 375). He might have been right if the type of theory he wanted had to be a more geometrical theory on the model of Maxwell’s electrodynamics and then GR grounded in Riemannian geometry. Such a theory was, he thought, unlikely to emerge from QM or QFT, and this may be true. Neither theory is, however, “a *description* of nature”, at least of the ultimate constitution of nature, because, at least in RWR interpretations (arguably on Einstein’s mind, given his debate with Bohr, of which this statement, more general as it is, is part), neither offers a representation of this constitution, which was Einstein’s imperative for a fundamental theory.

Einstein, who was no naïve realist, was aware that, just as GR was, a theory of the kind he wanted could only be a suitably mathematized *conceptual* idealization, the product of “a free conceptual construction” [37] (p. 47). Einstein would have also agreed with Dirac (who was in part inspired by Einstein’s “method” in GR) that “the most powerful method of advance” was that of proceeding from mathematics to physics, while, however, keeping realism in place. The main problem of the Heisenberg method for Einstein was not any lack of a free conceptual construction in Heisenberg’s theory but that this theory only provided the *algebra* of probabilities for the outcomes of quantum experiments, without a proper realist idealization, geometrical in character (even if it contained algebra, as relativity did), of the type provided by GR. Or so it appeared before singularities entered the theory within merely a year from the discovery of the theory with Schwarzschield’s 1915 work, although it took much longer to accept them as a permanent feature of the theory. Einstein never reconciled himself to the idea of singularities in relativity and thought they should ultimately be avoided. It is of course impossible to know how he would have responded to the current evidence and Roger Penrose’s 1965 proof (a decade after Einstein’s death in 1955) that singularities of black holes were a consequence of GR, without esoteric assumptions leading to singularities in previous solutions of Einstein’s equation of GR [38]. Penrose was awarded the 2020 Nobel prize in physics, which he shared with Andrea Ghez and Reinhard Genzel, who experimentally confirmed the existence of a massive black hole at the center of the Milky Way. It is true that black holes are ultimately quantum objects. This, however, does not help to mitigate their role in physically confirming the role of singularities by means of GR.

Einstein’s assessment of the Heisenberg method and QM requires qualifications, assigning this method greater complexity than Einstein’s statement conveys. This complexity also brings geometry back into the Heisenberg method and quantum theory. The Heisenberg method *was* fundamentally algebraic. It was also irreducibly probabilistic and allowed for RWR interpretations, primarily by virtue of the algebraic features of QM. This method, however, did not exclude geometry or topology, and it led to new developments in both fields, for example, in noncommutative geometry. Rather, QM brought with it a new way of geometrical thinking and a new, more abstract form of geometry, that of Hilbert spaces, of finite and infinite dimensions (over C) to fundamental physics. QM and QFT led to a new synergy of algebra and geometry in physics.

In saying that “we must give up, in principle, the space-time continuum”, Einstein must have had in mind the spacetime continuum in *representing* the ultimate physical reality by a geometrical theory, similarly to the way it was achieved by GR. Einstein was aware of the idea that this character may be discrete, an idea that had been around for long time by then. This possibility was considered by Riemann as early as 1854 in his Habilitation lecture, which Einstein knew [39]. Riemann brought this possibility up, on Kantian lines, in connection with the difference between our continuous phenomenal representation of physical space and “the reality underlying space”, which may be discrete [39] (p. 33). A subtler part of Riemann’s argument concerned the difference between the discrete and continuous nature of the reality underlying space in the infinitely small. If it is discrete, then the ground of the metric relation of the corresponding manifold would be given by the *mathematical* concept of this manifold itself. By contrast, if it is continuous, this ground would be defined by the physical principles arising from the physical phenomena considered. GR was a manifestation of the second case, as an example of both this grounding as such and a change in physical principles vis-à-vis those of Newton’s theory of gravity.

Quantum theory offered new reasons for the first possibility. The idea that “the reality underlying space” may be discrete has acquired additional currency in view of the difficulties of QFT, which are still around, the theory’s immense successes notwithstanding. The idea was, in view of these difficulties, advocated by, among others, Heisenberg from the 1930s on. His line of reasoning concerning it largely, albeit not entirely, faded away because of the successes of renormalization of QED in 1950 and then the electroweak theory in the 1970s. Nevertheless, the difficulties of QFT (self-interaction of elementary particles, vacuum polarization, and so forth) and, in view of the infinite values of quantities considered, the necessity of renormalization is seen as a problem by quite a few physicists and philosophers, beginning, as noted, with Dirac, the founder of QED. Richard Feynman, one of the inventors of the renormalizations of QED (for which he was awarded a Nobel prize), often reflected on these difficulties, including their bottom line:

It always bothers me that, according to the laws [of quantum theory] as we understand them today, it takes a computing machine an infinite number of logical operations to figure out what goes on in no matter how tiny a region of space, and no matter how tiny a region of time. How can all that be going on in that tiny space? Why should it take an infinite amount of logic to figure out what one tiny piece of space/time is going to do [40] (p. 57).

If space is discrete, this problem disappears. There is, however, no discrete mathematics thus far to handle quantum phenomena as QFT is able to as a mathematically continuous theory, its difficulties notwithstanding. As Feynman noted on the same occasion, the problem might also disappear if physics is no longer mathematical, without giving this eventuality much of a chance [40] (pp. 57–58). 

The idea of the ultimately discrete nature of “the reality underlying space” or physics beyond the current QFT scales is, while relatively marginal, very much alive. It has acquired new currency in attempts to reconcile QFT and GR, possibly by moving to a new type of theory (beyond quantum theory and relativity alike) that accounts for both quantum and gravitational phenomena. Among well-known proposals also aimed to offer alternatives to string and M-Brane theories (which are continuous) are the loop quantum gravity and causal network theory. 

A promising investigation supporting to the possibility that “the reality underling space” is discrete at the Planck scale was undertaken in [41,42] along the lines of quantum information theory. The authors derive both Maxwell’s theory and Dirac’s equation (in the absence of interactions) from discrete cellular–automata architecture, proceeding from a set of fundamental principles which require continuity. The spacetime continuum still enters the theory as required by both Maxwell’s equations and Dirac’s equation, but it becomes an emergent phenomenon. The Lorentz invariance and hence special relativity are broken at the Planck scale, while locality is retained, being defined by the assumption that physical systems can only be physically influenced by their immediate environment. The approach is also pertinent mathematically because of its use of geometric group theory. This theory emerged from the realization that discrete mathematical objects such as groups defined in algebraic terms can be considered as geometric-like objects and studied with geometric to topological techniques, developed for continuous mathematical entities. There are other theories of this type in mathematics, such as the Grothendieck’s étale topology discussed below. Still, while they have earlier precursors in algebra or even in geometry, such as finite geometries that began to be developed in the nineteenth century, these are recent developments. They have been competing with centuries or even millennia of the dominance of continuous thinking and mathematics based on it, which led to an immense multitude of rich and powerful theories. The very fact that these developments are modelled on continuous mathematics is a symptom of its dominance and an important point in considering the viability of discrete theories. 

By contrast, in (strong) RWR interpretations, QM or QFT do not require the assumption that the reality underlying space or the ultimate nature of physical reality is discrete. In these interpretations, the ultimate constitution of reality responsible for quantum phenomena is inconceivable and hence cannot be assumed to be discrete any more than continuous. If these interpretations of *quantum phenomena* themselves, which are strictly discrete relative to each other, are in place, it does not matter whether a theory predicting what is observed in these phenomena is mathematically continuous or discrete, insofar as RWR interpretations of this theory is possible. Continuity is a mathematical feature of the formalism of QM and QFT in their currently standard forms, just as it is that of the formalism of classical physics or relativity theory. Unlike in the latter theories, however, the continuous mathematics of QM and QFT relates, via the corresponding operator algebra (cum Born’s rule), to the data observed as discrete phenomena by predicting the probabilities or statistics of the occurrence of these data. At the same time, each quantum phenomenon, while discrete relative to any other, is described classically in the continuous space and time just as observed phenomena are in classical physics or relativity. Thus, the spacetime continuum is retained at this level. I leave aside the question of whether the continuity of space or time is ultimately only a feature of our phenomenality rather than of nature, even though the extension of the RWR view to all physics, which is possible, supports the idea that space and time belong to our phenomenal experience, as Kant argued (e.g., [1] (pp. vii–xxiv)).

On the one hand, these probabilistic relations between quantum theory and quantum phenomena are established via the algebra of Hilbert-space operators, which does offer the Heisenberg method its algebraic aspects. The continuous functions (over R) used in classical physics or relativity (for variables such as position or momentum) are replaced by operators and their algebra in Hilbert spaces over C. On the other hand, qualifying Einstein’s view, these Hilbert spaces themselves are continuous geometrical objects. They are defined analogously to mathematical spaces that represent physical spaces in classical physics and relativity, with the concept of distance (“norm” in Hilbert spaces) defined algebraically in both cases. Hilbert spaces offer one the possibility, often taken advantage of by physicists, to *think geometrically* by using both the rigor of algebraic formalism and our phenomenal geometrical intuition as heuristic help and guidance; in accord, “Think Geometrically, Prove Algebraically” [43]. The principle was introduced in algebraic geometry over finite fields (an important fact in the context of the relationships between algebra and geometry or discreteness and continuity), but it is equally applicable in QM and QFT [2] (pp. 76–83). QFT often deals with Hilbert spaces whose continuity is denser than that of regular continua such as the (real number) spacetime continuum of classical physics or relativity. In addition, continuous functions are retained because these Hilbert spaces are those of continuous functions, which are infinite-dimensional vectors when one is dealing with continuous variables such as “position” and “momentum” represented by operators. More accurately, these are abstract mathematical elements, *P* and *Q*, that allow one to predict the value as the position or the momentum (but never both together because of the uncertainty relations) observed in measuring instruments. In sum, rather than only algebraic, the Heisenberg method was also a new geometrical method in theoretical physics, bringing algebra and geometry together there. This method was adopted and developed by Dirac in founding QFT as, in Bohr’s words, “a most striking illustration of the power and fertility of the general quantum-mechanical way of description” [14] (v. 2, p. 64). 

Group theory, already central for physics, for example, in view of Noether’s theorems, became another crucial part of the algebraic mathematical machinery of quantum theory. This is because while quantum data can only be handled probabilistically, they also have a complex ordering by obeying various symmetry principles, including local symmetries. The latter are especially important in QFT, especially in leading to discoveries of new particles such as quarks and gluons inside the nucleus, eventually establishing the standard model of particle physics. When QFT predictions concern the effects associated with elementary particles of a given type, such as electrons, photons, and quarks (there are six types), the mathematics of prediction involves an irreducible representation of the corresponding symmetry. This is how Murrey Gell-Mann and Georges Zweig discovered quarks. They were considering the SU(3) flavor symmetry group for hadrons and noticed that no hadrons were associated with the irreducible representations or, as I think it is fitting to call them, “Galois atoms” of this group [2]. To assume that there were elementary particles associated with these representations implied, however, that hadrons were not elementary particles but composites of new particles, named quarks by Gell-Mann. 

Galois atoms *as a concept* (although not the term itself) were introduced by Eugene Wigner, whose work has a major importance in the history of symmetries and group representations in quantum theory, including as mathematically different from those in classical physics and relativity theory. This difference is in part due to the role of discrete symmetries such as mirror symmetries not associated with conservation laws, as some continuous symmetries are in accordance with Noether’s theorems. This is a testimony to both the role of abstract mathematics in quantum theory and to the immense reach of Galois’s concept of a group, far beyond the initial problem in algebra this concept was developed to address.

Eventually, Galois theory expressly entered QFT in the context of renormalization. While the role of Galois theory in renormalization is rarely reflected on beyond those who work on it, the subject of renormalization in general in QFT is extensively covered in physical and philosophical literature. Accordingly, I only summarize essential points most relevant in the present context. It was realized by the early 1930s that the computations provided by QED were reliable only to a first order of perturbation theory. Perturbation theory is a set of approximation methods using mathematical perturbation for describing a complex quantum system by using a simple one with a known solution and adding to it a “perturbing” Hamiltonian representing a small disturbance of that simple system. These computations led to the appearance of infinities when one attempted to use the formalism for calculations that would provide closer approximations matching the experimental data. These difficulties were eventually handled through the renormalization procedure, which became a crucial part of QFT and remains so, because QFT still contains these divergencies, specifically certain divergent and hence mathematically illegitimate integrals. Roughly speaking, the procedure manipulates such integrals by replacing them with finite integrals through artificial cut-offs at a certain stage of calculations. These cut-offs have no proper mathematical justification and are performed by putting in, by hand, experimentally obtained numbers that make these integrals finite, which removes the infinities (which happen to cancel each other) from the final results of calculations. The Yang–Mills theory is not renormalizable in dimensions higher than four, which does not affect the standard model the spacetime of which is four-dimensional. The renormalization of the corresponding Yang–Mills theories was crucial for both the electroweak unifications and quantum chromodynamics (QCD) (which handles strong interactions), two theories comprising the standard model.

At some point in this history, a new concept, that of the renormalization group, was introduced with the work of Kenneth G. Wilson arguably most significant in the development of its role. The renormalization group is a mathematical technique rather than a group in its technical sense (it may be seen as a semigroup), especially as part of the so-called effective QFTs that enable one to properly investigate the changes in a quantum system as represented (“viewed”) at different scales. It reflects the changes in the underlying force laws of QFT, changes due to the fact that the energy scale at which physical processes occur varies, with energy–momentum and resolution distance scales obeying the uncertainty relations. Actual computations are difficult and massively assisted by, and in fact are impossible without, the use of computers. Conceptually, one evaluates the transformations in the formalism in accordance with Feynman’s diagrams, which are, however, only heuristic guides to such calculations in most views, including Feynman’s own (e.g., [1], pp. 300–302).

Quite unexpectedly, Galois theory was brought up to recast the mathematics involved in a new form. The formal mathematical complexities are nearly prohibitive even for many working in QFT by virtue of using some stratospheric techniques, such as Grothendieck’s theory of motives, which would not be possible to consider here. My point here is the remarkable fact itself of the role of a “Galois group” in renormalization theory. The idea was initially suggested by Pierre Cartier, who designated the corresponding Galois group a “cosmic Galois group”, in view, one suspects, of its potential significance in the extension of QFT enabling one to handle gravity [44]. It is sufficient to consider the basic statement of how a specific (“motivic”) Galois group enters in Alain Connes and Matilde Marcolli’s article. The article and their related work also provide necessary formal definitions for a mathematically informed reader, as the mathematics is formidable and requires advanced levels of training. (A comprehensive exposition is offered in [45] (pp. 95–136), a long work, again, difficult technically.) They say:

The divergences of quantum field theory are a highly structured phenomenon [even beyond the renormalization group, which already structures them]. More precisely, they provide data that define an action of a specific “*motivic Galois group*” U* on the set of physical theories.

In particular, this exhibits the renormalization group as the action of a one-parameter subgroup Ga ⊂ U* of the above Galois group. …

The natural appearance of the “motivic Galois group” U* in the context of renormalization confirms a suggestion made by Cartier in [44] that in the Connes–Kreimer theory of perturbative renormalization one should find a hidden “cosmic Galois group” closely related in structure to the Grothendieck–Teichmüller group. The question of relations between the work of Connes–Kreimer, motivic Galois theory, and deformation quantization was further emphasized by Kontsevich in [46]. …

The “motivic Galois group” U* acts on the set of dimensionless coupling constants of physical theories, through the map of the corresponding group G to formal diffeomorphisms constructed in [47].

This also realizes the hope formulated in [48] of relating concretely the renormalization group to a Galois group. …

These facts altogether [all together?] indicate that the divergences of Quantum Field Theory, far from just being an unwanted nuisance, are a clear sign of the presence of totally unexpected symmetries of geometric origin [49]. (pp. 4073–4075)

This conclusion counters the common sentiments, some more strongly negative, including by Dirac, the founder of QED, toward the divergencies of QFT and then renormalization. These sentiments were tempered but far from vanquished by the successes of QFT enabled by renormalization, beginning with calculating the Lamb shift in the 1950s, by renormalizing QED by Sin-Itiro Tomonaga, Julian Schwinger, and Feynman, who were awarded the Nobel prize for this work. Even those who were less worried about the theory’s dependence on renormalization, such as Schwinger or Freeman Dyson, would not think of it as a reflection of deeper aspects of the mathematics of QFT or physics in high-energy quantum regimes.

This conclusion also suggests that the use of continuous mathematics in QFT may not be a problem that would *require* this mathematics to be replaced with discrete mathematics, thus making QFT a mathematically discrete theory at least at the deeper scales, ultimately the Planck scale. Instead, the continuous mathematics of QFT, including the very necessity of renormalization, *might be* a reflection of how nature or (a necessary qualification in RWR interpretations) our interactions with nature work in high-energy quantum regimes. Discrete alternatives cannot be excluded. As noted earlier, advocating them is understandable and, while not widespread, has been around for a long time, along with attempts to develop such alternatives. This, for the reasons noted earlier as well, is not an easy task, and most such attempts thus far tend to be based on transferring the methods of continuous mathematics to the discrete domain. This is not a criticism. Geometric group theory and Grothendieck étale-cohomology theory show the effectiveness of this approach in mathematics itself. It is perhaps not coincidental that the quantum-informational approach (by using quantum cellular automata) to QED based on discreteness mentioned above uses geometric groups theory [41,42]. As in dealing with other outstanding problems of fundamental physics, it is difficult to predict how this situation will be resolved, if it will ever be. On the other hand, “the symmetries of geometric origin” invoked by Connes and Marcolli do not appear to be in conflict with RWR interpretations of QFT. These interpretations may, however, apply to a discrete or finite theory that may replace QFT in its present form of a continuous theory, dealing with irreducibly discrete phenomena. It is important to keep in mind that, if found, such a theory is a theory and not the ultimate reality at stake in this theory, a reality that cannot be assumed to be discrete, any more than continuous, in RWR interpretations.

The understanding of the physical meaning and implications of the mathematics of the motivic Galois theory is a complex task that, as far as I know, have been barely pursued, in contrast to significant mathematical advances just described. What is remarkable is an unexpected and potentially far-reaching role of Galois theory in the mathematics of QFT, including in linking algebra and geometry there, just as they are sometimes linked by Galois theory in the mathematics of Riemann surfaces and then Grothendieck’s topos theory and étale cohomology, which led Grothendieck to motivic cohomologies (an introduction to motive theory in the set of contexts under discussion is offered in [50], yet another highly technical work). Certain purely mathematical aspects of Galois theory suggest that its role in QFT and renormalization group may not be so surprising. One can indeed formulate a general principle, the Galois principle, at work here.

First, one can think of the standard Galois theory in algebra as related to the “scales” corresponding to the fields extending the original field by a fundamental theorem of Galois theory. The theorem states that given a finite Galois extension *K/k*, there is a bijection between the set of subfields k⊂E⊂K and the subgroups H⊂K. Then, *E* is given by the set of invariants of *K* under the action of *H*, *E = K^H^ =* $\left\{a\in K:ga=a,g\in H\right\}$. If *H* is a normal subgroup, then *G/H* ≅ Gal (*E/k*), and conversely, if *E/k* is a normal field extension, then the corresponding subgroup Gal (*K/k*) is a normal group. In the case of the renormalization group, we deal with a transformation of theories rather than fields, but the principle remains in place. The corresponding Galois theory is metatheoretical when the domains governed by it are themselves theories, such as QFTs. *The Galois principle states that a given set of domains (fields, manifolds, or theories) is transformed by means of an algebraic structure, such as a group or semi-group (or some other structure) so that each domain is associated with a substructure of this structure, such as a subgroup by the Galois group of a field.* The connection between Galois theory and Riemann surfaces was established long before topos theory, and found, for example, in Weyl’s 1913 book on Riemann surfaces, known to J-P. Serre and possibly Grothendieck:

The group of cover transformations, regarded as an abstract group, expresses purely and completely everything in the relation between the normal covering surface $\overset{-}{F}$ and the base surface F which has the character of analysis situs [topology]. This group is also called the *Galois group* of $\overset{-}{F}$. It is in fact the analog of the Galois group of a normal algebraic field (of finite degree) over a base field [51] (p. 58).

Grothendieck’s key insight leading him to étale cohomology was to generalize, in terms of category theory, the concept of “open set”, beyond a subset of the algebraic variety (which offers only a very weak topology, Zariski’s topology, too weak to develop a proper cohomology theory over finite fields). This was possible because the concept of sheaf and of the cohomology of sheaves could be defined by any category rather than only that of open sets of a given space. I bypass the technical definition of the key concepts discussed, such as those of category, sheaf, cohomology, or topos, which are not germane here (they are discussed in detail in [2]). QM have been considered and in fact recast in terms of category theory, but as discussed in [1] (pp. 307–328), virtually exclusively in terms of this recasting, without revealing new physical or epistemological features of quantum theory, let alone developing new theories, as it was by Grothendieck in algebraic geometry. Étale cohomology is defined by this type of replacement, specifically by using the category of étale mappings of an algebraic variety, which become “open subsets” of the finite unbranched covering spaces of the variety, a vast and radical generalization of Riemann’s concept of a covering space. Part of the genealogy of this generalization was the fact that the fundamental group of a topological space, say, again, a Riemann surface, could be defined in two ways. First, it can be defined geometrically, as the group of equivalence classes of the sets of all loops at a given point, with the equivalence relation given by homotopy. Alternatively, it can be defined as a group of transpositions of covering spaces. In this second definition, it is analogous to the Galois group of the algebraic closure of a field, thus confirming the genealogy of étale cohomology in both Riemann’s and Galois’s work. An étale mapping offers a sufficient number of open sets to define adequate cohomology groups for some coefficients for an algebraic variety over a finite field. In the case of complex varieties, one recovers the standard cohomology groups with coefficients in any constructible sheaf.

This is an example, in mathematics itself, of “the mathematical complexity principle”, stating that if it is discrete (including finite), a theory has to have a structural complexity analogous and related to that of the continuous mathematical theory used in the domain considered, a principle that I extend to quantum theory with some help from Grothendieck. As noted above, there are similar developments in contemporary mathematics such as in geometric groups theory. In fact, already discrete and finite geometries may be seen as defined by assigning the discrete the complexity akin to that of continuous. One might accordingly expect a similar situation in the case of discrete theories in physics, if they are to handle the same or even more complex phenomena currently handled by QFT. 

The category of étale mappings is what is called a topos, a concept that is, for now, the most abstract algebraic form of spatiality in mathematics. It was seen as such by Grothendieck himself, who compared his contribution to the idea of space in mathematics to that of Einstein in GR in physics and that of Schrödinger [52] (pp. 68). A reference to Einstein and GR, which transformed our understanding of spatiality in physics, via Riemann, whom Grothendieck invokes as well, makes immediate sense. On the other hand, a reference to Schrödinger is not self-evident and may even appear strange, unless Grothendieck refers to Schrödinger’s work on GR (which, while possible, seems unlikely) rather than on QM. In fact, it makes, or (I am not sure that Grothendieck had it in mind) can be given, a sense. As explained in this article, QM established an entirely new type of relationship, fundamentally probabilistic in nature, between purely mathematical spaces, Hilbert spaces over C, in Schrödinger’s case infinite-dimensional ones, and the actual (three-dimensional) physical space, mathematically represented as a real manifold. Technically, it was Heisenberg (not mentioned by Grothendieck) who first defined this architecture of QM, while Schrödinger initially thought that the wave function would represent quantum processes, as wave-processes, in space and time. Schrödinger, as noted, never reconciled himself with the (RWR) view of Heisenberg and Bohr and came to see QM as merely a convenient method of (probabilistic) calculations rather than as a proper fundamental theory [32] (p. 167) [1] (pp. 145–166).

The argument of this article also questions or at least qualifies Grothendieck’s “prediction” made on the same occasion. This prediction concerns the “expected renewal” (“if it must yet come”) of our understanding of the ultimate nature of spatiality or, by implication, physical reality, a prediction that was made by Grothendieck in reflecting on Riemann’s insight that “the reality underlying space” may be discreet. On the other hand, this argument allows for Riemann’s view or (they are not the same) Grothendieck’s conjecture concerning the nature of this reality itself and for formulating the mathematical complexity principle in quantum theory. (The following comments build on [2,4], which, however, do not discuss the mathematical complexity principle, my main focus here.) According to Grothendieck,

It must be already fifteen or twenty years ago that, leafing through the modest volume constituting the complete works of Riemann, I was struck by a remark of his “in passing”. He pointed out that it could well be that the ultimate structure of space is discrete, while the continuous representations that we make of it constitute perhaps a simplification (perhaps excessive, in the long run...) *of a more complex reality*; That for the human mind, “the continuous” was easier to grasp than “the discontinuous”, and that it serves us, therefore, as an “approximation” to apprehend the discontinuous.

This is a remark of a surprising penetration in the mouth of a mathematician, at a time when the Euclidean model of physical space had never yet been questioned; in the strictly logical sense, it is rather the discontinuous which traditionally served as a mode of technical approach to the continuous.

Mathematical developments of recent decades have, moreover, shown a much more intimate symbiosis between continuous and discontinuous structures than was imagined, even in the first half of this century.

In any case finding a “satisfactory” model (or, if necessary, a set of such models, “satisfactorily connecting” to each other) of “continuous”, “discrete”, or of “mixed” nature—such work will surely involve a great conceptual imagination, and a consummate flair for apprehending and unveiling new types of mathematical structures.

This kind of imagination or “flair” seems rare to me, not only among physicists (where Einstein and Schrödinger seem to have been among the rare exceptions), but even among mathematicians (and here I speak with full knowledge).

To summarize, I predict that the expected renewal (if it must yet come) will come from a mathematician in soul well informed about the great problems of physics, rather than from a physicist. But above all, it will take a man with “philosophical openness” to grasp the crux of the problem. This is by no means a technical one but rather a fundamental problem of natural philosophy ([52] (p. 67–68); cited in [53] (pp. 164, 195)).

One could not really say that “the Euclidean model of physical space had never yet been questioned” at the time of Riemann’s lecture in 1854, given that non-Euclidean geometry and with it the view that physical space may not be Euclidean was around for two decades by then. Also, “Riemann’s remark” was not “in passing”. It was germane to his argument (e.g., [2] (pp. 118–132)]. One must, of course, keep in mind Grothendieck’s quotation marks and the fact that this is a recollection, occasioning Grothendieck’s own view, rather than an analysis of Riemann’s argument. 

Grothendieck’s insight itself, however, which reverses the conventional relationships between continuity and discontinuity, is profound. As explained above, his greatest work in algebraic geometry was grounded in extending the cohomological architecture of algebraic topology developed for continuous manifolds to discrete algebraic varieties via his concepts of a scheme and a topos. More significant for the present article are, however, implications of Grothendieck’s reversal of the standard relationships between the discrete and the continuous, the possibility of which in physics bears especially on QFT. As discussed above, in QFT, the continuity of space or time leads to difficulties not found in QM, difficulties that have made the view that “the reality underlying space” might be discrete to have periodically reemerged from the rise of QFT to our own time. On the other hand, QFT is a viable theory in handling all other fundamental forces of nature even if not in the way many would prefer, especially in view of the necessity of renormalization. There is, thus far, no effective discrete theory for doing so.

As such, QFT allows one to *give* a clear sense to the point stated by Grothendieck for “the human mind” (where this point is less certain and is not claimed by Riemann) to the effect that “‘the continuous’… serves… as an ‘approximation’ to apprehend the discontinuous”. As discussed earlier, some believe that continuous (high-energy) QFTs may only be approximations of discontinuous ones, which should apply at the higher energy scale, ultimately at the Planck scale. Even if this does not prove to be the case and we continue to use a mathematically continuous theory, such as QFT, this view is consistent and reasonable on physical grounds. It is also in accord with Riemann’s argument, which is physical, concerning the discrete reality underlying space [2], (pp. 118–131). Riemann’s statements concerning the subject there clearly show that the way Riemann sees the situation or what he says is different from what Grothendieck assumes in his comments (keeping in mind that one deals with Grothendieck’s recollection rather than a reading of Riemann). It is possible that Riemann *thought* along the lines stated by Grothendieck. Riemann’s actual point in the Habilitation lecture was, however, that the reality underlying space or, by implication, the ultimate constitution of physical reality may be either discrete or continuous (in the immeasurably small), and that the determination of this reality is different as a result. This difference is as follows: “in a discrete manifold the principle of metric relations is already contained in the concept of the manifold, but in a continuous one it must come from something else”, namely from physical rather than merely mathematical considerations [39] (p. 33). 

“The continuous representations that we make of [the discrete structure of space] constitute perhaps a simplification (perhaps excessive, in the long run...) *of a more complex reality*”, Grothendieck says, thus suggesting that the discrete may be more complex than the continuous. Grothendieck does not explain why this may be the case. On the other hand, in RWR interpretations (assuming that the ultimate nature of physics is quantum), the ultimate constitution of reality is beyond the reach of thought: hence this constitution is neither continuous nor discontinuous, any more than conforming to any concept we can form. As such, this reality may be “a more complex reality”, more complex than any we can imagine, literally speaking. The assumption that a reality is beyond thought allows this reality to be *unimaginably* (literally speaking!) complex. Indeed, it is likely that, limited by our evolutionary biological and neurological nature, we can imagine very little of how nature ultimately is, if the word “to be” applies, and the same may be true about how thought itself is, including in dealing with mathematical reality. 

There is no evidence that Riemann ever contemplated this type of reality, which is not surprising given that quantum theory was half a century away, with no indication that anything like it could come about. Nor does it appear that Grothendieck had ever considered this type of reality either, even if without accepting it. His invocation of Einstein and Schrödinger, rather than Bohr or Heisenberg, suggests that he would have been unlikely to accept this type of conception, assumed by Bohr or Heisenberg. Nor does Grothendieck appear to have contemplated an RWR-type interpretation of mathematical reality, although some of his ideas suggest such an interpretation [2] (pp. 175–179). On the other hand, his insight into the reversal or the more complex relationship between the continuous and discontinuous is profound, inviting me to apply the mathematical complexity principle to fundamental physics.

A more fundamental theory underlying QFT (SQFT) and possibly avoiding infinities of QFT or even containing quantum gravity may be discrete, thus making the continuous mathematical character of QFT approximate the discrete or discontinuous character of this underlying theory (SQFT may or may not be a “quantum theory”, as we understand the term now). If so, however, SQFT has to have at least as much or greater mathematical complexity than our continuous fundamental theories such as QFT. We do not have such a discrete theory for now; in any event, not beyond hypothetical proposals, few of which appear to be likely to succeed, although some of them, such as a theory of quantum cellular automata in [41,42] mentioned above, hold, I think, a greater promise, and are in accord with the mathematical complexity principle. I suspect, however, still using Heisenberg’s method, combining geometry and algebra, and continuous and discrete, that such a theory is more likely to be mixed. It is also likely that, as Grothendieck said, “finding a ‘satisfactory’ model (or, if necessary, a set of such models, ‘satisfactorily connecting’ to each other) of ‘continuous’, ‘discrete’ or of ‘mixed’ nature—such work will surely involve a great conceptual imagination”.

On the other hand, one might doubt Grothendieck’s final “prediction”: “I predict that the expected renewal (if it must yet come) will come from a mathematician in soul well informed about the great problems of physics, rather than from a physicist”. History suggests otherwise. Things were more mixed early with Descartes and Newton, both of whom were physicists as well as mathematicians. It is also true that Riemann and Gauss as well as a few other mathematicians made major contributions to physics. Among major examples are Euler, D’Alembert, Laplace, or Hamilton, or in the case of expressly modern mathematics Hilbert, von Neumann, and Weyl, or quite a few others, some (admittedly a limited sample) mentioned in this article, throughout the twentieth and twenty-first century. These contributions were, however, primarily mathematical in nature, unlike Gauss’s and Riemann’s work in physics. Both, of course, also made mathematical contributions to physics. The work I am referring to now, however, was *physics*, which, it is also true, had in turn a shaping influence on their mathematics, certainly in the case of Riemann who works on several expressly physical projects. Still, when it comes to breakthroughs and renewals, especially by the time physics began to use modern mathematics, they mostly came from physicists: Galileo, Newton, Maxwell, Boltzmann, Planck, Einstein, Bohr, Heisenberg, and Dirac, to name only some of the key figures. It took Einstein and his GR to offer rigorous physical content to Riemann’s insights by bringing together the physics of gravitation and Riemannian geometry, even though Riemann, “the genius of Riemann, solitary and uncomprehended”, was credited by Einstein [54] (p. 190). Einstein’s characterization itself is not quite accurate: Riemann and his genius were neither entirely solitary nor entirely uncomprehended by his contemporaries(Gauss, for one) unlike, say, Galois and his genius. In any event, it was Einstein who discovered the physics in question and realized that it needed a certain new type of mathematics to be handled, even though he was helped by his friend Marcel Grossman, a mathematician, in finding and mastering this mathematics.

Quantum theory is an entirely different story. While some of the key ideas existing in mathematics earlier were used, and while mathematicians, such as Weyl and von Neumann, importantly contributed, primarily mathematically, to quantum theory, the credit for the *creation* of quantum theory belongs strictly to physicists, beginning with Planck and Bohr. Both Heisenberg and Dirac have reinvented some already available mathematics and created new ones. As my argument in this article suggests, given the history of modern physics (which is my Bayesian prior), solving the main outstanding problems of fundamental physics, whatever degree of “renewal” this requires, is more likely to come from a physicist or several physicists, admittedly greatly informed mathematically, than from a mathematician. On the other hand, one might be hesitant to predict the origins of mathematics necessary to deal with these problems, that is, whether this mathematics will emerge in physics, as in Heisenberg’s discovery of QM, or in mathematics already in place, as in Einstein’s discovery of GR by using Riemannian geometry. One might, again, conversely, obtain help from physics in dealing with the difficult problems of mathematics, as in Simon Donaldson’s use of the Yang–Mills theory in the topology of four-dimensional manifolds. The Connes–Marcolli collaboration is another important example of the productive symbiosis of mathematical and physical thinking. I do not argue otherwise here. I argue, however, that this help is limited because one still needs to assign this mathematics the full measure of *mathematics*. In both cases, mathematics ultimately carries the day in physics as a mathematical–experimental science, with mathematics defining this conjunction.

This view may justify Grothendieck’s claim differently. Grothendieck is speaking of an individual imagination in dealing with such problems in physics. It is, again, of some interest that, while granting a potential primacy to the discontinuous, he invokes an example of the necessary “kind of imagination and ‘flair’”—Einstein and Schrödinger. Both were thinkers of continuity, who, as discussed in this article, tried to avoid the discontinuous in physical reality, even if with imagination and flair. Would one not need instead the kind of imagination and flair for the discontinuous found in Heisenberg and Bohr? A safer bet, which I suggest here and which Grothendieck might have shared, would be that it is still *mathematics* that will move physics forward, as it has accomplished throughout the history of modern physics as mathematical–experimental science, with mathematics, again, defining this conjunction. In this sense, a physicist who would be able to do so would be a mathematician, “a mathematician in soul”, thus confirming Grothendieck’s prediction, even if not in the way Grothendieck had in mind.

Heisenberg, this article argues, was a physicist who was such a mathematician in soul. His thinking leading him to his invention QM established an entirely new type of relationship between continuity and discontinuity, which also correlatively redefined the relationships, which are my main concern in this article, between mathematics and physics, especially modern mathematics and modern physics, bringing about what Bohr saw as “the new era” of their mutual stimulation [14] (v. 1, p. 51). First, these relationships (combining both kinds just mentioned) were between the *mathematical continuity* of the formalism of QM and the *physical discontinuity* of quantum phenomena, always discrete relative to each other. Second, epistemologically more radically, he abandoned the assumption that QM must represent the continuous (and causal) connections between these discrete phenomena and, in the first place, the assumption of the existence of such connections. These assumptions grounded all preceding physical theories, such as classical physics and relativity theory, and made them realist. Instead, the ultimate reality responsible for quantum phenomena was assumed to be beyond representation and eventually (courtesy of Bohr’s interpretation) beyond conception, designated here as a “reality without realism”, RWR, which makes the way quantum phenomena come about real but beyond the reach of thought, and thus literally *un*-thinkable. 

Grothendieck was, however, right to think that “above all, it will take a man with ‘philosophical openness’ to grasp the crux of the problem. This is by no means a technical one but rather a fundamental problem of natural philosophy”. It might also be a woman like Emmy Noether, a key figure in the genealogy of Grothendieck’s own mathematics, in which she appears by name in the important concept of a Noetherian scheme. When it comes to discontinuity, Heisenberg, especially at the time of his invention of QM, and Bohr would, again, be better examples, certainly in their philosophical openness to the discontinuous, in a manifested juxtaposition to Einstein and Schrödinger, as thinkers of continuity. It is not a matter of avoiding continuity, or correlatively geometry, in the first case, or discontinuity, or correlatively algebra, in the second, which was not the aim of these figures and is arguably impossible. It is a matter of a relative balance and the vision of the ultimate nature of reality and the means by which we relate to it. As a result, with quantum physics, modern physics also acquired a new type of relationship among the discrete, the continuous, and the unthinkable. Still, as in all modern physics, from Descartes and Galileo on, it was a *mathematical* project, a new type of mathematical project initiated by Heisenberg, that brought about this entirely new situation in physics. 

## Data Availability

Data is contained within the article.
